# Is meeting 24-hour movement guidelines associated with a lower risk of frailty among adults?

**DOI:** 10.1186/s12966-025-01722-x

**Published:** 2025-02-21

**Authors:** Yuhang Liu, Siyao Gao, Zhigang Dou, Zhen Chen, Jialing Tang

**Affiliations:** 1 School of Physical Education and Sports, Central China Normal University, Wuhan, 430079 P. R. China; 2 Department of Physical Education, Central South University, Changsha, 410083 P. R. China; 3 Henan Experimental High School, Zhengzhou, 450002 P. R. China

**Keywords:** 24-hour movement guidelines, Frailty, Age disparities, Gender disparities, Racial disparities, Adults, NHANES

## Abstract

**Background:**

The relationship between 24-hour (24-h) movement guidelines and frailty remains unclear. We aimed to investigate the associations between meeting 24-h movement guidelines and frailty and to conduct secondary analyses by age, gender, and ethnicity.

**Methods:**

In this study, we extracted data from the National Health and Nutrition Examination Survey (2007–2018) database. A total of 5,726 participants (50.25 ± 0.31 years) were included in this analysis, representing 38,240,356 noninstitutionalized U.S. individuals. Three 24-h movement behaviors, namely, physical activity, sedentary behavior (SB), and sleep, were self-reported using the standardized questionnaires. The 49-item frailty index was used to measure frailty. Multivariable logistic regression models and trend tests were used to examine the associations between meeting 24-h movement guidelines and frailty. Sensitivity analyses were also conducted to ensure the robustness of our results.

**Results:**

The total age-adjusted prevalence of frailty was 30.5%. In the fully adjusted model, compared with not meeting any of the 24-h movement guidelines, the adjusted odds ratios (AORs) of frailty were 0.786 (95% CI: 0.545, 1.133), 1.161 (95% CI: 0.787, 1.711), and 0.915 (95% CI: 0.616, 1.358) for participants meeting only moderate to vigorous physical activity (MVPA), only SB, and only sleep guidelines, respectively, but no statistically significant differences were observed (all *P* values > 0.05). Participants who met the SB + sleep guidelines (AOR = 0.613, 95% CI: 0.423, 0.887), MVPA + sleep guidelines (AOR = 0.389, 95% CI: 0.255, 0.593), and MVPA + SB guidelines (AOR = 0.555, 95% CI: 0.383, 0.806) presented a significantly lower risk of frailty by 39%, 61%, and 45%, respectively. Meeting all 3 guidelines (AOR = 0.377, 95% CI: 0.264, 0.539) and meeting 2 guidelines (AOR = 0.527, 95% CI: 0.377, 0.736) were associated with a lower risk of frailty (*P* value < 0.001), showing a linear trend (*P* for trend < 0.001). The strength of these associations varied somewhat by age, gender, and ethnicity.

**Conclusions:**

Significant associations between the 24-h movement guidelines and frailty were observed among U.S. adults. Future studies are warranted to examine the causality and trajectory of these associations.

**Supplementary Information:**

The online version contains supplementary material available at 10.1186/s12966-025-01722-x.

## Background

There is no doubt that frailty is one of the most serious public health issues [1]. Frailty is characterized by the accumulation of health defects stemming from the impairment of health caused by physical, physiological, and psychological factors [2, 3]. This condition is closely associated with adverse outcomes, including mortality, disability, hospitalization, cognitive decline, and lower quality of life [4]. Individuals with frailty are more likely to present with multimorbidity (≥ 2 diseases) [5], imposing a heavy burden on society and the health care system [6]. Frailty affects millions of older adults globally, and the impact of frailty is expected to continue to increase as the population ages [4].

The severity of frailty is dynamic, and it could be improved through the implementation of nutritional supplementation and physical exercise programs [7]. Therefore, identifying modifiable risk factors is crucial to guide public health and prevent frailty. The associations between several modifiable risk factors related to lifestyle, such as physical activity (PA), sedentary behavior (SB), sleep, diet pattern, and frailty, have been well documented [8–11]. For example, Pond and colleagues reported that each minute of PA was associated with better frailty status in individuals with diabetes mellitus (DM) and highlighted the importance of replacing SB time with PA to improve frailty status [9]. Song et al. reported a strong negative association between more sedentary time and incident physical frailty [10]. Nakakubo et al. reported that sleeping 6 h or less, or 9 h or more, was associated with a greater likelihood of becoming frail [11]. However, most previous studies have focused on the separate effects of one movement behavior on frailty. From a holistic perspective, the joint effects of typical 24-hour (24-h) movement behaviors (PA, SB, and sleep) were comparatively ignored [12]. Recently, a growing body of evidence has supported the combined effects of these movement behaviors on health outcomes [13, 14]. Moreover, based on time-use epidemiology and empirical findings [15], in October 2020, the Canadian 24-hour (24-h) movement guidelines for adults aged 18–64 years were released [16]. The guidelines recommend that adults aged 18–64 years should engage in a minimum of 150 min of moderate to vigorous physical activity (MVPA) per week, limit SB time to no more than 8 h daily, and ensure 7–9 h of high-quality sleep per night. Importantly, the guidelines highlight the importance of movement behaviors throughout the whole day (24-hour period) for health outcomes because engagement in one behavior inevitably impacts the allocation of time to the other two behaviors [16, 17].

The launch of the 24-h movement guidelines has inspired relevant research interest. Recent studies have reported that adhering to the 24-h movement guidelines positively influences several health outcomes among children, adolescents, and adults. For example, an investigation from Canada revealed that, compared with adults who met 2 or fewer recommendations, those who met al.l 3 guidelines had better body mass index (BMI), waist circumference, aerobic fitness, and cardiometabolic values [18]. Another systematic review indicated that meeting 24-h movement guidelines was significantly associated with skeletal health, health-related quality of life, and psychosocial health [12, 19]. However, to the best of our knowledge, the relationship between 24-h movement guidelines and frailty among adults has not been examined.

Considering the evidence on the links between meeting 24-h movement guidelines and health indicators, the 24-h movement guidelines, as a comprehensive and modifiable strategy, may have great potential in preventing and relieving frailty. Information on how 24-h movement behaviors are potentially associated with frailty could be used to inform multicomponent interventions. In addition, previous research has reported differences in the prevalence of frailty and adherence to 24-h movement guidelines by age, gender, and ethnicity [4, 20]. Therefore, based on the National Health and Nutritional Examination Survey (NHANES), we aimed to investigate the associations of meeting 24-h movement guidelines with frailty in U.S. adults and to conduct secondary analyses by age, gender, and ethnicity.

## Methods

This cross-sectional study was conducted and reported following the criteria outlined in the Strengthening the Reporting of Observational Studies in Epidemiology statement [21].

### Study design and participants

The NHANES, a continuous nationwide survey initiated in 1999, is conducted biennially by the National Center for Health Statistics (NCHS) and the Centers for Disease Control and Prevention to investigate health-related behaviors, socioeconomic and nutritional status, and the physical examination results of the general public. The NHANES researchers employed a complex, stratified, multistage sampling design to secure a nationally representative sample of noninstitutionalized U.S. civilians. The NCHS Ethics Review Board approved all NHANES protocols, and all study participants provided written informed consent. The detailed description and datasets of the NHANES can be found at http://www.cdc.gov/nchs/nhanes/about_nhanes.htm. As NHANES data are free for public use and available online, consent from a medical ethics committee is not required [22].

**Fig. 1:**
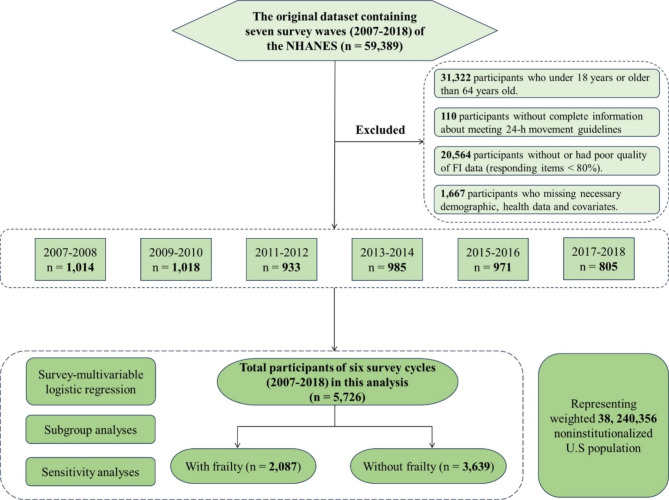
Flowchart of selection of participants. Abbreviations: NHANES = national health and nutrition examination survey; FI = frailty index

In the present study, we extracted data from six survey cycles of the NHANES (2007–2008, 2009–2010, 2011–2012, 2013–2014, 2015–2016, and 2017–2018). Initially, 59,389 participants were enrolled in this study. Following the Canadian 24-Hour Movement Guidelines for Adults aged 18–64 years, individuals younger than 18 years or older than 64 years were excluded (*n* = 31,322). Participants without complete information on 24-h movement guidelines (*n* = 110), who had poor frailty index (FI) assessments (*n* = 20,564), or who lacked the necessary covariate information (*n* = 1,667) were also excluded from this analysis. The selection process for participants is shown in Fig. 1.

### Measurement of meeting 24-h movement guidelines

The Canadian 24-hour movement guidelines for adults aged 18–64 years included MVPA, SB time, and sleep duration. Data on self-reported moderate to vigorous physical activity (MVPA) and sedentary behavior (SB) in the NHANES were obtained from the Global Physical Activity Questionnaire [23], which has been widely utilized globally, and its validity and reliability have been demonstrated across various populations [24]. The participants were asked how many days of MVPA (e.g., moderate working activities, recreational activities) they typically engaged in a week, and further asked the amount of time they spent performing MVPA (vigorous-intensity work activities, recreational activities) each day [25]. Individuals who accumulated ≥ 150 min/week of MVPA (1 min of vigorous PA equating to 2 min of moderate PA) were considered to meet the PA guidelines. SB is defined as activities that do not increase energy expenditure beyond rest (< 1.5 MET), such as watching television, working on a computer, lying and sitting during waking hours, and engaging in other forms of screen-based entertainment [26]. The SB time was calculated through self-reported data on the PAD 680: question “How much time do you usually spend sitting on a typical day?” (https://wwwn.cdc.gov/Nchs/Nhanes/2017-2018/P_PAQ.htm#PAD680). Sleep duration was self-reported by participants. The sleep duration data were derived from the SLD010H question: “How much sleep do you get (hours)?” (https://wwwn.cdc.gov/Nchs/Nhanes/2013-2014/SLQ_H.htm#SLD010H) from 2007 to 2014, and the SLD012 question: “How much sleep do you usually get at night on weekdays or workdays?” (https://wwwn.cdc.gov/Nchs/Nhanes/2017-2018/SLQ_J.htm#SLD012 ) from 2015 to 2018.

### Ascertainment of frailty

Frailty was assessed using the FI developed by Hakeem et al., on the basis of the standard procedure introduced by Searle and colleagues [27, 28]. The FI includes 49 diagnostic items that cover cognition (1 item), dependence (15 items), depressive symptoms (7 items), comorbidities (13 items), hospital utilization and access to care (5 items), physical performance and anthropometry (2 items), and laboratory values (6 items). Each entry is evaluated according to adjudication criteria, which allot a score within a range of 0 (no defect present) to 1 (most severe defect). The FI is subsequently derived by dividing the cumulative score by the total number of items responded to. To ensure the quality of frailty diagnoses, individuals who responded to at least 80% of 49 items (approximately 40) were included in this analysis. It has been reported that if a sufficient number of items are responded to, they could be randomly selected and still yield similar results for the risk of poor outcomes [29]. The cut-off point of the FI was 0.21 [30], and participants with an FI greater than or equal to 0.21 were considered frail. The detailed scoring criteria are presented in Supplementary Table 1.

### Assessment of covariates

The covariates included sociodemographic variables [(age, gender, ethnicity, education level, marital status, and the poverty income ratio (PIR)], lifestyle variables [(BMI, smoking status, alcohol consumption, Healthy Eating Index-2015 (HEI-2015), and total energy intake)], and health-related variables: hypertension, cardiovascular diseases (CVDs), and DM.

Age was divided into 3 groups: 18–39 years, 40–59 years, and 60–64 years. Ethnicity was classified into 4 categories: non-Hispanic White, non-Hispanic Black, Mexican American, and other races. Education levels were classified into 3 categories: less than high school, high school, and more than high school. The participants were classified according to their marital status, as widowed/divorced/separated, never married, or married/living with their partner. The PIR was categorized as < 1.3, 1.3–3.5, or > 3.5. BMI was categorized into 4 groups: underweight (< 18.5 kg/m^2^), normal (18.5–25 kg/m^2^), overweight (25–30 kg/m^2^), and obese (≥ 30 kg/m^2^). Both smoking and alcohol consumption status were categorized into 3 groups: never, former, and current. Total energy intake and HEI-2015 scores were obtained through 24-hour dietary interviews. The HEI-2015 is a comprehensive indicator for evaluating dietary quality, with scores ranging from 0 to 100, with higher scores reflecting better diet quality. The diagnosis of hypertension was established based on the average of 3 consecutive tests, with a systolic pressure ≥ 140 mmHg and a diastolic pressure ≥ 90 mmHg. The presence of CVDs (coronary heart disease, myocardial infarction, stroke, or angina) was determined by participants receiving these diagnoses from a health professional before the survey. DM was defined as fasting plasma glucose ≥ 126 mg/dL, 2-h plasma glucose ≥ 200 mg/dL, hemoglobin A1c ≥ 6.5%, or self-reported diabetes diagnosed by a professional doctor.

### Statistical analysis

We followed the analytical guidelines and survey weights recommended by the NHANES in this analysis [31]. To compare the characteristics between the frail and non-frail populations, we employed t-tests to compare continuous variables [presented as weighted mean ± standard error (SE)] and chi-squared tests for categorical variables (reported as counts and weighted percentages). Multivariable weighted logistic regression models were used to evaluate the adjusted odds ratios (AOR) and 95% confidence intervals (CI) for the associations of meeting 24-h movement guidelines (none, MVPA, SB, sleep, MVPA + SB, MVPA + sleep, and SB + sleep), the number of guidelines met (none, 1, 2, and 3) with frailty status (“yes” or “no”). We presented the results of these associations in the same table and conducted multivariable weighted logistic regressions separately for each association. The reference group for all comparisons is set as participants who did not adhere to any of the 24-h movement guidelines. A total of three models were constructed. The crude model was unadjusted. Model 1 was adjusted for age, gender, and ethnicity. Based on Model 1, we further adjusted for more demographic, lifestyle, and health-related variables (education level, marital status, the PIR, BMI, smoking status, alcohol consumption status, and total energy intake) into Model 2 to increase the accuracy of our results. Trend tests were performed to investigate the linear trends between the number of guidelines met and frailty. We also conducted analyses stratified by gender, age, and ethnicity, and the results are presented separately for each group. To ensure the robustness of our results, we conducted two sensitivity analyses: (1) additional adjustments accounting for the survey cycle, HEI-2015, CVDs, hypertension, and DM in Model 2, respectively; and (2) modification of the frailty diagnostic criteria to the threshold of 0.25 [32]. In addition, we investigated the associations of FI (continuous variable) with 24-h movement guidelines. All the statistical analyses were performed using R language (X64 Version 4.3.1, R Foundation for Statistical Computing). A two-tailed *P*-value of < 0.05 was considered to indicate statistical significance across all analyses.

## Results

### Characteristics of the study participants

A total of 5,726 participants were included in this analysis from six survey waves ranging from 2007 to 2018, representing 38,240,356 noninstitutionalized U.S. individuals. The characteristics of the participants stratified by frailty status are summarized in Table 1. The weighted mean age of the sample was 50.25 ± 0.31 (mean ± SE) years, with 52.4% (*n =* 2,956) being females. The majority of participants were non-Hispanic White (71.4%, *n* = 2,435), followed by non-Hispanic Black (11.3%, *n* = 1,382), other races (11.3%, *n* = 1,131), and Mexican American (5.9%, *n* = 252). More than half of the participants (57.1%, *n* = 2,785) had more than a high school education, and approximately two-thirds of the participants were overweight (29.5%, *n* = 1,701) or obese (46.5%, *n* = 2,719). Approximately 60% of the sample (*n* = 3,231) were married or living with parents, whereas 45.8% (*n* = 2,570) and 73.5% (*n* = 3,876) of the respondents were never smokers and now drinking alcohol, respectively. The participants without frailty were likely to be male, non-Hispanic white, married/living with parents, have a higher education level, have a higher PIR, be nonsmokers, and have no chronic diseases (all *P* values < 0.05). The characteristics of the participants stratified by meeting the 24-h movement guidelines and the number of guidelines met are displayed in Supplementary Tables 2 and 3, and the characteristics of the participants stratified by age, sex, and ethnicity are presented in Supplementary Tables 4–6.

**Table 1:** Survey-weighted baseline characteristics of study participants stratified by frailty

	Prevalence of age-adjusted frailty [(weighted, % (SE)]	Estimate U.S population (*n*)	Total participants	Frailty		*P*-value^a^
				Yes	No	
**Overall**	30.5 (1.04)	38,240,356	5,726 (100%)	2,087 (31.4)	3,639 (68.6)	-
**Age**	-	-	50.25 ± 0.31	50.81 ± 0.31	49.99 ± 0.42	0.12
18–39	-	8,363,840	1,090 (21.9)	295 (16.0)	795 (24.6)	**< 0.001**
40–59	-	15,909,506	2,170 (41.6)	1,146 (60.7)	1,024(32.9)	
60–64	-	13,967,010	2,466 (36.5)	646 (23.3)	1,820 (42.6)	
**Gender**						
Female	36.7 (1.37)	20,029,120	2,956 (52.4)	1,245 (62.0)	1,711 (48.0)	**< 0.001**
Male	24.0 (1.24)	18,211,236	2,770 (47.6)	842 (38.0)	1,928 (52.0)	
**Race/ethnicity**						
Non-Hispanic White	28.3 (1.42)	27,320,808	2,435 (71.4)	875 (65.1)	1,560 (74.4)	**< 0.001**
Non-Hispanic Black	42.7 (1.98)	43,26,130	1,382 (11.3)	585 (16.2)	797 (9.1)	
Mexican American	26.9 (2.02)	2,254,032	778 (5.9)	252 (5.7)	526 (6.0)	
Other Races	34.4 (2.08)	4,339,386	1,131 (11.3)	375 (13.0)	756 (10.6)	
**Education level**						
Less than high school	47.3 (4.30)	1,837,285	569 (4.8)	249 (7.4)	320 (3.6)	**< 0.001**
High school	36.0 (1.51)	14,577,396	2,372 (38.1)	990 (46.2)	1,382 (34.4)	
More than high school	25.4 (1.18)	21,825,675	2,785 (57.1)	848 (46.3)	1,937 (62.0)	
**PIR**	-	-	2.75 ± 0.05	1.99 ± 0.06	3.09 ± 0.06	**< 0.001**
<1.3	45.7 (1.37)	11,359,008	2,394 (29.7)	1,194 (46.6)	1,200 (22.0)	**< 0.001**
1.3–3.5	29.6 (1.42)	12,306,713	1,871 (32.2)	634 (33.0)	1,237 (31.8)	
>3.5	17.1 (1.78)	14,574,635	1,461 (38.1)	259 (20.4)	1,202 (46.2)	
**BMI (kg/m** ^**2**^ **)**	-	-	30.60 ± 0.16	33.13 ± 0.27	29.44 ± 0.17	**< 0.001**
Underweight (< 18.5)	26.6 (4.19)	720,798	99 (1.9)	39 (1.8)	60 (1.9)	**< 0.001**
Normal (18.5–25)	23.1 (1.68)	8,404,260	1,207 (22.0)	316 (15.4)	891 (25.0)	
Overweight (25–30)	26.8 (1.83)	11,281,329	1,701 (29.5)	509 (24.2)	1,192 (31.9)	
Obese (≥ 30)	37.1 (1.42)	17,833,970	2,719 (46.6)	1,223 (58.6)	1,496 (41.1)	
**Marital status**						
Married/Living with Partner	28.4 (1.44)	23,289,542	3,231 (60.9)	1,035 (54.4)	2,196 (63.9)	**< 0.001**
Never married	29.0 (2.04)	6,444,753	969 (16.9)	330 (14.7)	639 (17.9)	
Widowed/Divorced/Separated	45.3 (2.80)	8,506,060	1,526 (22.2)	722 (30.9)	804 (18.3)	
**Smoking status**						
Never	22.3 (1.04)	17,514,971	2,570 (45.8)	750 (33.3)	1,820 (51.5)	**< 0.001**
Former	31.3 (2.14)	10,122,100	1,464 (26.5)	533 (26.4)	931 (26.5)	
Current	41.8 (1.74)	10,603,285	1,692 (27.7)	804 (40.3)	888 (22.0)	
**Alcohol consumption**						
Never	27.5 (2.88)	3,910,625	746 (10.2)	237 (8.7)	509 (10.9)	**< 0.001**
Former	44.5 (2.62)	6,224,614	1,104 (16.3)	558 (25.1)	546 (12.3)	
Current	28.1 (1.08)	28,105,118	3,876 (73.5)	1,292 (66.3)	2,584 (76.8)	
**Hypertension**						
No	20.1 (1.05)	19,136,353	2,612 (50.0)	644 (32.4)	1,968 (58.1)	**< 0.001**
Yes	45.8 (1.76)	19,104,003	3,114 (50.0)	1,443 (67.6)	1,671 (41.9)	
**DM**						
No	24.7 (1.04)	27,392,181	3,780 (71.6)	1,098 (56.4)	2,682 (78.6)	**< 0.001**
Yes	50.4 (2.06)	10,848,175	1,946 (28.4)	957 (21.4)	989 (43.6)	
**CVDs**						
No	26.4 (0.99)	33,531,879	4,894 (87.7)	1,487 (73.9)	3,407 (94.0)	**< 0.001**
Yes	64.3 (2.97)	4,708,477	832 (12.3)	600 (26.1)	232 (6.0)	
**SB time (Minutes/per day)**	-	-	381.59 ± 4.11	394.56 ± 6.78	375.64 ± 4.88	**< 0.05**
**Sleep time**	-	-	7.06 ± 0.03	6.84 ± 0.06	7.17 ± 0.04	**< 0.001**
**Total MVPA (Minutes/per week)**	-	-	1,175.71 ± 41.54	1,036.45 ± 79.21	1,224.42 ± 47.40	**< 0.05**
**Total energy intake (kcal)**	-	-	2,147.56 ± 16.93	2,009.50 ± 26.90	2,210.82 ± 21.36	**< 0.001**
**Meeting 24-h movement guidelines**						
None	47.9 (3.46)	3,267,676	524 (8.5)	295 (13.4)	229 (6.3)	**< 0.001**
**Meeting individual guidelines**						
MVPA	33.2 (3.10)	2,690,772	386 (7.0)	150 (7.6)	236 (6.8)	
SB	50.8 (3.43)	3,509,966	718 (9.2)	398 (15.4)	320 (6.3)	
Sleep	37.2 (3.21)	3,912,983	534 (10.2)	234 (13.0)	300 (8.9)	
**Meeting specific guideline combinations**						
SB + Sleep	32.9 (2.55)	4,619,359	762 (12.1)	269 (12.8)	493 (11.7)	
MVPA + Sleep	28.5 (1.97)	6,606,322	495 (11.8)	99 (6.2)	396 (14.4)	
MVPA + SB	17.7 (2.10)	4,513,279	1,035 (17.3)	354 (16.5)	681 (17.6)	
**Number of guidelines met**						
0	47.9 (3.46)	3,267,676	524 (8.5)	295 (13.4)	229 (6.3)	**< 0.001**
1	41.0 (1.98)	10,113,722	1,638 (26.4)	782 (36.0)	856 (22.1)	
2	26.5 (1.31)	15,738,960	2,292 (41.2)	722 (35.5)	1,570 (43.8)	
3	20.5 (1.39)	9,119,998	1,272 (23.8)	288 (15.0)	984 (27.9)	
**Year cycle**						
2007–2008	31.9 (2.98)	5,737,947	1,014 (15.0)	375 (15.3)	639 (14.9)	0.5
2009–2010	30.0 (2.10)	5,626,439	1,018 (14.7)	377 (14.5)	641 (14.8)	
2011–2012	28.8 (2.93)	6,751,423	933 (17.7)	331 (15.9)	602 (18.5)	
2013–2014	33.3 (2.06)	6,907,893	985 (18.1)	380 (20.0)	605 (17.2)	
2015–2016	29.7 (2.26)	7,072,092	971 (18.5)	359 (19.1)	612 (18.2)	
2017–2018	29.4 (2.06)	6,144,562	805 (16.1)	265 (15.3)	540 (16.4)	

Continuous variables are presented as weighted mean ± SE, and categorical variables are presented as n (weighted %)

^a^*P*-values were assessed by T-test (continuous variables) or by Chi-square test (categorical variables). *P*-values shown in bold were statistically significant.

Abbreviations: BMI = body mass index; CVDs = cardiovascular diseases; DM = diabetes mellitus; MVPA = moderate-to-vigorous physical activity; PIR = poverty income ratio; SB = sedentary behavior; SE = standard error

### Prevalence of adherence to the 24-h movement guidelines

Overall, 386 (7.0%), 718 (9.2%), and 534 (10.2%) participants met only the MVPA guidelines, only the SB guidelines, and only the sleep guidelines, respectively. Approximately one-tenth of the participants (8.5%, *n* = 524) met none of the 3 movement guidelines, whereas a quarter of the participants (23.8%, *n* = 1,272) followed all 3 movement guidelines. Approximately 40% of the participants met 2 of the 3 guidelines (*n* = 2,292), whereas approximately one-fifth (17.3%, *n* = 1,035) met the MVPA + SB guidelines. The total age-adjusted prevalence of frailty was 30.5%. The participants who met 0, 1, 2, and all 3 of the 24-h movement guidelines, with the age-adjusted prevalence of frailty, were 47.9%, 41.0%, 26.5%, and 20.5%, respectively (Table 1).

### Association of meeting individual guidelines with frailty

**Fig. 2:**
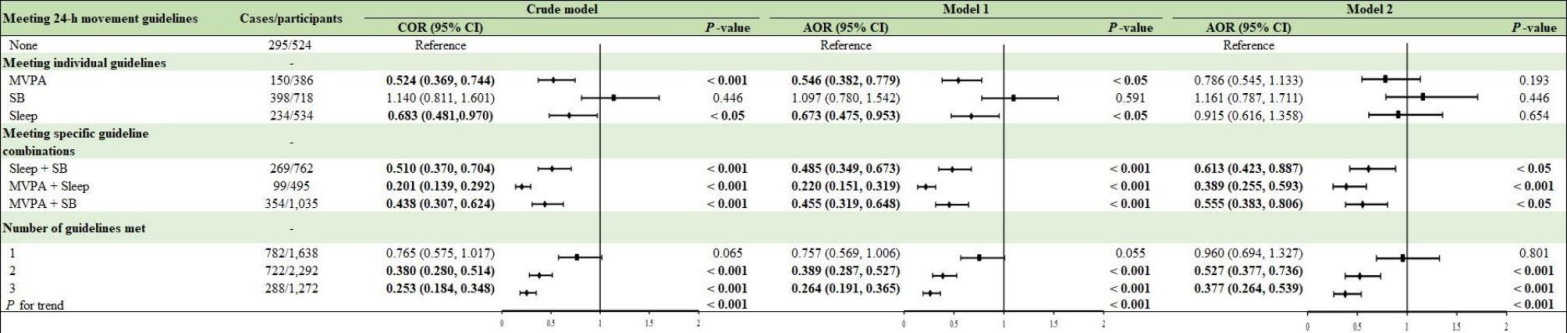
Associations of meeting 24-h movement guidelines with frailty. The results of COR (95% CI), AOR (95% CI), and *P*-value shown in bold were statistically significant. Model 1 was adjusted for age, gender, race/ethnicity. Model 2 was additionally adjusted for education level, marital status, PIR, BMI, smoking, alcohol consumption and total energy intake. AOR = adjusted odds ratio; BMI = body mass index; CI = confidence interval; COR = crude odds ratio; MVPA = moderate-to-vigorous physical activity; PIR = poverty income ratio; SB = sedentary behavior

As shown in Fig. 2, compared with participants who did not meet any of the 3 guidelines, those who adhered to either the MVPA [crude model: (crude odds ratios) COR = 0.524, 95% CI: 0.368, 0.744; Model 1: AOR = 0.546, 95% CI: 0.382, 0.779] or sleep (crude model: COR = 0.683, 95% CI: 0.481, 0.970; Model 1: AOR = 0.673, 95% CI: 0.475, 0.953) guidelines presented a lower likelihood of frailty. In the fully adjusted model (Model 2), the AOR of frailty was 0.786 (95% CI: 0.545, 1.133), 1.161 (95% CI: 0.787, 1.711), and 0.915 (95% CI: 0.616, 1.358) for participants who met only MVPA, only SB, or only sleep guidelines, respectively (All *P* values > 0.05). Furthermore, the associations between meeting individual guidelines and frailty stratified by age, gender, and ethnicity were similar, and not found to be statistically significant after adjusting for relative covariates (Tables 2, 3 and 4).

**Table 2:** Associations of meeting 24-h movement guidelines with frailty stratified by age

Meeting 24-h movement guidelines	Cases/participants	Age 18–39 years (n = 1,090)		Cases/participants	Age 40–59 years (n = 2,170)		Cases/participants	Age 60–64 years (n = 2,466)	
		AOR (95% CI)	*P*-value		AOR (95% CI)	*P*-value		AOR (95% CI)	*P*-value
None	44/88	Reference		150/228	Reference		101/208	Reference	
**Meeting individual guidelines**	-			-			-		
MVPA	28/94	0.681 (0.290, 1.598)	0.371	84/153	0.836 (0.471,1.483)	0.535	38/139	0.679 (0.334,1.379)	0.279
SB	51/98	1.190 (0.520, 2.721)	0.677	227/319	1.114 (0.660,1.880)	0.681	120/301	1.049 (0.619,1.779)	0.857
Sleep	18/65	0.605 (0.274, 1.334)	0.209	125/217	0.874 (0.503,1.519)	0.629	91/252	1.095 (0.569,2.107)	0.784
**Meeting specific guideline combinations**	-			-			-		
Sleep + SB	18/87	**0.400 (0.166, 0.965)**	**< 0.05**	165/281	0.910 (0.495,1.674)	0.76	86/394	**0.467 (0.257,0.850)**	**< 0.05**
MVPA + Sleep	19/116	0.495 (0.201, 1.224)	0.126	53/166	**0.393 (0.219,0.705)**	**< 0.05**	27/213	**0.268 (0.123,0.587)**	**< 0.05**
MVPA + SB	68/253	**0.398 (0.182, 0.872)**	**< 0.05**	186/388	**0.518 (0.305,0.879)**	**< 0.05**	100/394	0.808 (0.431,1.513)	0.499
**Number of guidelines met**	-			-			-		
1	97/257	0.811 (0.407, 1.615)	0.546	463/689	0.945 (0.603,1.481)	0.802	249/692	0.983 (0.582,1.659)	0.948
2	105/456	**0.421 (0.201, 0.879)**	**< 0.05**	404/835	**0.573 (0.347,0.947)**	**< 0.05**	213/1,001	**0.523 (0.309,0.885)**	**< 0.05**
3	49/289	**0.282 (0.140, 0.570)**	**< 0.001**	156/418	**0.413 (0.237,0.722)**	**< 0.05**	83/565	**0.432 (0.233,0.801)**	**< 0.05**
*P* for trend			**< 0.001**			**< 0.001**			**< 0.001**

The results of AOR (95% CI), and *P*-value shown in bold were statistically significant. The multivariable model was adjusted for gender, race/ethnicity, education level, marital status, PIR, BMI, smoking, alcohol consumption and total energy intake. Abbreviations: AOR = adjusted odds ratio; BMI = body mass index; CI = confidence interval; MVPA = moderate-to-vigorous physical activity; PIR = poverty income ratio; SB = sedentary behavior

**Table 3:** Associations of meeting 24-h movement guidelines with frailty stratified by gender

Meeting 24-h movement guidelines	Cases/participants	Female (n = 2,956)				Cases/participants	Male (n = 2,770)			
		Crude model		Adjusted Model			Crude model		Adjusted Model	
		COR (95% CI)	*P*-value	AOR (95% CI)	*P*-value		COR (95% CI)	*P*-value	AOR (95% CI)	*P*-value
None	169/279	Reference		Reference		126/245	Reference		Reference	
**Meeting individual guidelines**	-					-				
MVPA	94/181	0.702 (0.418, 1.180)	0.179	0.981 (0.547, 1.758)	0.947	56/205	**0.411 (0.246, 0.687)**	**< 0.001**	0.614 (0.358, 1.054)	0.076
SB	251/427	1.037 (0.664, 1.619)	0.871	1.048 (0.629, 1.746)	0.856	147/291	1.246 (0.782, 1.985)	0.351	1.314 (0.781, 2.211)	0.3
Sleep	152/326	0.597 (0.369, 0.968)	0.037	0.877 (0.507, 1.517)	0.635	82/208	0.759 (0.443, 1.301)	0.312	0.948 (0.569, 1.581)	0.837
**Meeting specific guideline combinations**	-					-				
Sleep + SB	170/459	**0.397 (0.262, 0.602)**	**< 0.001**	**0.520 (0.328, 0.823)**	**< 0.05**	99/303	0.685 (0.421, 1.114)	0.125	0.803 (0.469, 1.374)	0.418
MVPA + Sleep	55/234	**0.183 (0.107, 0.312)**	**< 0.001**	**0.342 (0.198, 0.592)**	**< 0.001**	44/261	**0.243 (0.148, 0.398)**	**< 0.001**	**0.450 (0.259, 0.781)**	**< 0.05**
MVPA + SB	193/459	**0.458 (0.297, 0.706)**	**< 0.001**	**0.529 (0.322, 0.867)**	**< 0.05**	161/576	**0.448 (0.283, 0.710)**	**< 0.001**	**0.581 (0.363, 0.930)**	**< 0.05**
**Number of guidelines met**	-					-				
1	497/934	0.755 (0.510, 1.119)	0.159	0.954 (0.605, 1.506)	0.839	285/704	0.755 (0.507, 1.126)	0.166	0.950 (0.622, 1.452)	0.812
2	418/1,152	**0.355 (0.244, 0.515)**	**< 0.001**	**0.477 (0.315, 0.724)**	**< 0.001**	304/1,140	**0.421 (0.284, 0.624)**	**< 0.001**	**0.594 (0.386, 0.914)**	**< 0.05**
3	161/591	**0.224 (0.141, 0.357)**	**< 0.001**	**0.345 (0.211, 0.564)**	**< 0.001**	127/681	**0.305 (0.196, 0.473)**	**< 0.001**	**0.418 (0.245, 0.713)**	**< 0.05**
*P* for trend			**< 0.001**		**< 0.001**			**< 0.001**		**< 0.001**

The results of COR (95% CI), AOR (95% CI), and *P*-value shown in bold were statistically significant. Adjusted Model was adjusted for age and race/ethnicity, education level, marital status, PIR, BMI, smoking, alcohol consumption and total energy intake. Abbreviations: AOR = adjusted odds ratio; BMI = body mass index; CI = confidence interval; MVPA = moderate-to-vigorous physical activity; PIR = poverty income ratio; SB = sedentary behavior

### Association of meeting specific guideline combinations with frailty

Compared with participants who met none of the guidelines, those who adhered to SB + sleep guidelines (Model 2: AOR = 0.613, 95% CI: 0.423, 0.887), MVPA + sleep guidelines (Model 2: AOR = 0.389, 95% CI: 0.255, 0.593), and MVPA + SB guidelines (Model 2: AOR = 0.555, 95% CI: 0.383, 0.806) had a significantly decreased risk of frailty (Fig. 2). The associations of meeting specific guideline combinations with frailty, stratified by age, gender, and ethnicity, and not adhering to any 24-h movement guidelines was set as the reference, are presented in Tables 2, 3 and 4. First, regarding age group differences, no significant associations were observed between adherence to MVPA + sleep (18–39 years), SB + sleep (18–39 years), MVPA + SB guidelines (40–64 years), and frailty (all *P*-value > 0.05). Second, in males, meeting the SB + sleep guidelines (Model 2: AOR = 0.803, 95% CI: 0.469, 1.374) was not significantly associated with frailty after controlling for the covariates. Finally, the associations between meeting specific guideline combinations and frailty were stronger among the non-Hispanic White population compared to non-Hispanic black, Mexican American, and other races.

### Association of the number of guidelines met with frailty

When comparing frailty status between groups with different counts of 24-h movement guidelines met, participants who met all 3 guidelines (Model 2: AOR = 0.377, 95% CI: 0.264, 0.539; *P* value < 0.001) and those who met 2 guidelines (Model 2: AOR = 0.527, 95% CI: 0.377, 0.736; *P* value < 0.001) had a significantly lower likelihood of frailty (Fig. 2). The relationship between meeting only 1 guideline and a reduced risk of frailty was not statistically significant (Model 2: AOR = 1.076, 95% CI: 0.816, 1.407; *P* value > 0.05). The trend test revealed significant results in all age groups (Table 2; all *P* values for trend < 0.001), and this effect was more pronounced in individuals aged 18–39 years than in those in the other two age groups. As shown in Table 3, the number of guidelines met was significantly associated with a lower risk of frailty in both men and women. In addition, when this association was stratified by ethnicity, a significant inverse relationship between the number of guidelines met and the risk of frailty was also observed, except for in Mexican Americans (Model 2: AOR = 0.421, 95% CI: 0.175, 1.013) (Table 4).

**Table 4:** Associations of meeting 24-h movement guidelines with frailty stratified by race

Meeting 24-h movement guidelines	Cases/participants	Non-Hispanic White (n = 2,435)		Cases/participants	Non-Hispanic Black (n = 1,382)		Cases/participants	Mexican American (n = 778)		Cases/participants	Other Races (n = 1,131)	
		AOR (95% CI)	*P*-value		AOR (95% CI)	*P*-value		AOR (95% CI)	*P*-value		AOR (95% CI)	*P*-value
None	145/242	Reference		83/149	Reference		20/40	Reference		47/93	Reference	
**Meeting individual guidelines**	-			-			-			-		
MVPA	62/172	0.773 (0.471, 1.270)	0.305	56/126	0.710 (0.372, 1.355)	0.293	30-Jul	0.483 (0.131, 1.784)	0.265	20/58	1.450 (0.561, 3.752)	0.438
SB	153/242	1.141 (0.627, 2.074)	0.662	109/208	1.161 (0.697, 1.935)	0.559	65/124	1.364 (0.505, 3.680)	0.529	71/144	1.238 (0.642, 2.384)	0.519
Sleep	105/257	0.816 (0.476, 1.397)	0.453	68/131	0.989 (0.566, 1.729)	0.97	26/58	1.066 (0.404, 2.814)	0.894	35/88	1.585 (0.583, 4.312)	0.362
**Meeting specific guideline combinations**	-			-			-			-		
Sleep + SB	90/271	**0.567 (0.326, 0.985)**	**< 0.05**	74/167	0.704 (0.422, 1.177)	0.177	45/154	0.490 (0.210, 1.145)	0.097	60/170	1.052 (0.501, 2.209)	0.891
MVPA + Sleep	55/281	**0.392 (0.227, 0.676)**	**< 0.05**	23/87	**0.442 (0.234, 0.834)**	**< 0.05**	May-37	0.470 (0.204, 1.082)	0.074	16/90	0.453 (0.178, 1.151)	0.095
MVPA + SB	140/416	**0.509 (0.297, 0.873)**	**< 0.05**	104/282	0.671 (0.413, 1.090)	0.105	40/131	**0.176 (0.045, 0.695)**	**< 0.05**	70/206	0.712 (0.353, 1.433)	0.336
**Number of guidelines met**	-			-			-			-		
1	325/671	0.895 (0.570, 1.404)	0.624	233/465	0.957 (0.596, 1.535)	0.852	98/212	1.063 (0.433, 2.608)	0.891	126/290	1.379 (0.719, 2.645)	0.328
2	285/968	**0.489 (0.304, 0.787)**	**< 0.05**	201/536	**0.639 (0.414, 0.986)**	**< 0.05**	90/322	**0.417 (0.191, 0.909)**	**< 0.05**	146/466	0.767 (0.406, 1.449)	0.408
3	120/554	**0.404 (0.248, 0.659)**	**< 0.001**	68/232	**0.379 (0.220, 0.651)**	**< 0.001**	40/204	0.421 (0.175, 1.013)	0.053	56/282	**0.270 (0.130, 0.563)**	**< 0.001**
*P* for trend			**< 0.001**			**< 0.001**			**< 0.001**			**< 0.001**

The results of AOR (95% CI), and P-value shown in bold were statistically significant. The multivariable model was adjusted for age, gender, education level, marital status, PIR, BMI, smoking, alcohol consumption and total energy intake. Abbreviations: AOR, Adjusted odds ratio; BMI, Body mass index; CI, Confidence interval; MVPA, Moderate-to-vigorous physical activity; PIR, Poverty income ratio; SB, Sedentary behavior

### Sensitivity analyses

To ensure the robustness of our findings, sensitivity analyses were conducted. First, we additionally adjusted for the survey cycle, the HEI-2015 scores, CVDs, hypertension, and DM in Model 2, respectively (Supplementary Table 7), and further modified the diagnostic criteria of frailty from 0.21 to 0.25 (Fig. 2). The results of these supplementary analyses were generally in line with the primary analysis to support the strength and reliability of the observed associations (Supplementary Fig. 1). Specifically, when the diagnostic threshold value of frailty was 0.25, a statistically significant association was observed between only meeting MVPA guidelines and a lower risk of frailty, and the AOR of frailty was much lower (Model 2: AOR = 0.279, 95%CI: 0.187, 0.416) for participants who met all 3 guidelines than for those who met none of the 3 guidelines. We also examined the associations of the frailty index (continuous variable) with 24-h movement guidelines, which is consistent with our main results. In addition, when the FI was the outcome, significant relationships between only met the MVPA guideline (Model 2: *β*= -0.031, 95% CI: -0.049, -0.014), only met sleep guideline (Model 2: *β*= -0.029, 95% CI: -0.045, -0.013), and FI were detected (Supplementary Table 8).

## Discussion

### Main findings

The main findings of this study can be condensed into the following points: (1) adherence to any of the MVPA, SB, or sleep guidelines alone was not significantly associated with a reduced risk of frailty; (2) meeting 2 guidelines was significantly associated with a lower likelihood of frailty, and there were differences in the associations according to age, gender and ethnicity. Notably, meeting the MVPA and sleep guidelines in combination had the strongest negative association with frailty; and (3) compared with participants who did not meet any guidelines, individuals who met all three 24-h movement guidelines had a significantly lower risk of frailty. Specifically, as the number of 24-h movement guidelines met increased, the risk for frailty decreased. However, among Mexican Americans, there was no statistically significant difference between meeting all 3 guidelines and frailty risk. After sensitivity analyses were performed, our results remained stable. Our study broadens the knowledge regarding the importance of 24-h movement behaviors on frailty among general adults.

### Comparison with prior studies

The present study revealed that meeting only one of three guidelines was not significantly associated with a lower AOR of frailty. When the diagnostic threshold value of frailty was adjusted to 0.25, we observed a reduction in frailty risk for individuals who met only the MVPA guidelines, which corroborates the consensus to adhere to the World Health Organization recommendation of 150 min of MVPA per week for the prevention of non-communicable diseases [33]. A systematic review and meta-analysis that included 10 cohort studies showed that a higher level of PA was associated with decreased risks of frailty, and even stratified by PA measurement tools, produced a similar protective effect [34]. However, the relationships of adherence solely to SB guidelines or sleep guidelines with the risk of frailty were not fully consistent with previous related works. A study using NHANES data revealed that frail individuals were highly sedentary and that SB was associated with frailty in middle-aged to older adults [35]. A Mendelian randomization study demonstrated that SB (especially watching television) inversely affected frailty, suggesting potential biological heterogeneity behind specific sedentary activities [36]. Thus, the different types of sedentary activities may influence this association between SB guidelines and frailty, which might partly result in mixed results. A study from Mexico showed that, over a 4.4-year follow-up period, individuals who slept ≤ 5 h or ≥ 9 h presented a significantly increased risk of frailty [37]. Salinas-Rodríguez et al. reported that individuals with a moderate/poor stable sleep trajectory had greater odds of frailty than those with a very-good increasing trajectory [38]. Therefore, sleep quality may largely contribute to the inconsistent results of the association between only meeting sleep guidelines and frailty. Overall, meeting only one of the 24-h movement guidelines, while ignoring the other two, had limited impacts on frailty.

Regarding specific guideline combinations and frailty, our results showed a negative association between meeting any two of three guidelines and frailty. However, the associations varied by age, gender, and ethnicity to some extent. For meeting the MVPA + SB guidelines, we found that adhering to the combined guidelines for MVPA and SB was associated with a reduced risk of frailty, which is generally consistent with the existing evidence [39–41]. However, this significant association was not apparent in individuals aged 60 to 64 years. Research has shown that frailty is an extreme consequence of the normal aging process [4]. Thus, according to the geroscience hypothesis, this difference may be partly explained by the aging process decreasing the thresholds necessary for disease-specific insults to result in overt pathology [42]. For meeting SB + sleep guidelines in combination, our results revealed that adhering to the combined guidelines for SB + sleep was significantly correlated with a lower risk of frailty. A Mendelian randomization study suggested that genetic predispositions towards increased television viewing and diurnal napping were positively correlated with the FI, whereas sleep duration was inversely associated with the FI [43]. Compared with males, females generally have more SB time and experience greater sleeping disturbances [44, 45]. Therefore, the potential benefits of adhering to the combined guidelines for SB + sleep in mitigating frailty may be more sensitive for females [46]. The origin of the sex differences in this association needs to be further investigated in future studies. Notably, we observed the strongest relationship between adhering to the combined guidelines for MVPA + sleep and frailty. Based on the current evidence [47], one possible explanation for this association is the bidirectional relationship between sleep and PA. A study that examined cross-lagged time series of sleep and PA over 14 days and nights found that better sleep quality can lead to more PA the next day [48], and physical activity is recognized to maintain or improve the function of many physiological systems, including muscle and cardiac function, cognition, endocrine system, and inflammation, thereby improving frailty status [49]. Additionally, the ethnicity disparity in relationships may be partly explained by the imbalance in the number of people included in this study among the various ethnic groups.

Previous studies have demonstrated the close relationship between frailty and chronic diseases, such as CVDs, chronic kidney diseases, and chronic obstructive pulmonary diseases [50–52]. Moreover, the coexistence of frailty and multimorbidity is often observed [53]. The present study suggested that adherence to 24-hour movement guidelines is associated with lower odds of frailty, which is in line with previous related studies. As depicted in the systematic reviews, children who met the 24-h movement guidelines demonstrated better health-related quality, better cognition, and greater aerobic fitness [12]. Kastelic et al. reported that the likelihood of better self-rated health increased with the number of guidelines met among adults [54]. Furthermore, prior research revealed that adherence to all three 24-h movement guidelines was significantly associated with physical (muscle, adiposity), mental (depression, cognitive performance), and metabolic health [55–59], which are closely related to the content of the FI. Therefore, it is not surprising that the number of 24-h movement guidelines met is associated with a lower risk of frailty in adults.

### Implications

This study has important implications for public health and practice. First, our findings revealed a significant association between 24-h movement guidelines and frailty. This result highlights the importance of improving adults’ daily movement behaviors (MVPA, SB, and sleep) in frailty prevention. Second, secondary analyses suggest that there may be optimal combinations of 24-h movement guidelines for preventing frailty among the different sociodemographic groups. Moreover, adherence to the counts of guidelines was inversely associated with the prevalence of frailty. This means that in cases where the 24-h movement guidelines cannot be fully adhered to, individuals may strategically follow specific combinations of 24-h movement guidelines, thereby gaining relatively substantial health benefits. Third, these findings provide valuable insights for policy-makers in the development of frailty prevention strategies, and health professionals may advise that improving lifestyles may help frailty prevention. Finally, further high-quality studies are needed to validate the gender, age, and ethnicity differences in the associations of 24-h movement guidelines with frailty, and to identify optimal combinations of these guidelines to efficiently manage frailty risk when people cannot fully adhere to the 24-h movement guidelines in daily lives.

### Strengths and limitations

To the best of our knowledge, this is the first study to examine the relationship between meeting the 24-h movement guidelines and frailty. We also explored the age, gender, and ethnicity disparities in this relationship. This work addresses the evidence gaps and provides basic lifestyle recommendations for protecting against frailty in adults. Additional strengths include the nationally representative sample, and the adjustment for several potential confounders, such as CVDs, diet quality, hypertension, and DM, which allowed our findings to be generalizable at the population level. However, some limitations should be noted. First, because of the cross-sectional design, we cannot determine the causal relationship between meeting 24-h movement guidelines and a lower risk of frailty. Second, the data of all three 24-h movement behaviors were collected with self-reported items. Although these measures have been well validated in previous investigations, the findings of our study may have been affected by recall errors and social desirability bias. Future studies are warranted to validate these findings using objectively measured data. Third, the sample sizes in the secondary analyses of the associations between meeting 24-h movement guidelines and frailty were relatively small. Further studies are needed to validate the observed associations with larger sample sizes. Finally, despite our best efforts to select potential confounders, our analysis may still be affected by unmeasured or insufficiently measured confounders.

## Conclusions

In summary, our study indicated that the number of guidelines met was significantly associated with a lower risk of frailty. Following any combination of two guidelines may have benefits in reducing the risk of frailty. However, there may be differences in this association according to age, gender, and ethnicity. For a single guideline, meeting the MVPA guideline has a stronger positive association with frailty than the other two guidelines. Future research is warranted to examine the causality of these associations, and to employ a longitudinal study design to investigate the trajectories of how meeting the 24-h movement guidelines affects frailty progression.

## Electronic supplementary material

Below is the link to the electronic supplementary material.

**Supplementary Material 1**: **Supplementary Fig. 1** Associations of meeting 24-h movement guidelines with frailty (the cut of point = 0.25). **Supplementary Table 1** Variables in the 49-item frailty index and their respective scorings. **Supplementary Table 2** Baseline characteristics of study participants stratified by meeting 24-h movement guidelines. **Supplementary Table 3** Baseline characteristics of study participants stratified by the number of guidelines met. **Supplementary Table 4** Baseline characteristics of study participants stratified by age. **Supplementary Table 5** Baseline characteristics of study participants stratified by gender. **Supplementary Table 6** Baseline characteristics of study participants stratified by race/ethnicity. **Supplementary Table 7** Associations of meeting 24-h movement guidelines with frailty index (continuous variable). **Supplementary Table 8** Further adjustments in sensitivity analyses for associations of meeting 24-h movement guidelines with frailty

**Supplementary Material 2**: STROBE checklist

**Supplementary Material 3**: Graphical abstract

## Data Availability

The data could be collected from openly available resources at https://www.cdc.gov/nchs/nhanes/index.htm.

## Abbreviations

AOR: Adjusted odds ratios
BMI: Body mass index
CI: Confidence intervals
CVDs: Cardiovascular diseases
COR: Crude odds ratio
DM: Diabetes mellitus
FI: Frailty index
HEI-2015: Healthy eating index-2015
MVPA: Moderate to vigorous physical activity
NCHS: National Center for Health Statistics
NHANES: National Health and Nutritional Examination Survey
PA: Physical activity
PIR: Poverty income ratio
SB: Sedentary behavior
SE: Standard error
24-h: 24-hour

## References

[1] Dent E, Martin FC, Bergman H, Woo J, Romero-Ortuno R, Walston JD. Management of frailty: opportunities, challenges, and future directions. Lancet. 2019;394(10206):1376–86. 10.1016/S0140-6736(19)31785-4. 31609229 10.1016/S0140-6736(19)31785-4

[2] Hubbard RE, Theou O. Frailty: enhancing the known knowns. Age Ageing. 2012;41(5):574–5. 10.1093/ageing/afs093. 22780984 10.1093/ageing/afs093

[3] Liu Y, Han Y, Gao Y, et al. The association between oxidative balance score and frailty in adults across a wide age spectrum: NHANES 2007–2018. Food Funct. 2024;15(9):5041–9. 10.1039/d4fo00870g. 38651948 10.1039/d4fo00870g

[4] Hoogendijk EO, Afilalo J, Ensrud KE, Kowal P, Onder G, Fried LP. Frailty: implications for clinical practice and public health. Lancet. 2019;394(10206):1365–75. 10.1016/S0140-6736(19)31786-6. 31609228 10.1016/S0140-6736(19)31786-6

[5] Hanlon P, Nicholl BI, Jani BD, Lee D, McQueenie R, Mair FS. Frailty and pre-frailty in middle-aged and older adults and its association with multimorbidity and mortality: a prospective analysis of 493 737 UK Biobank participants. Lancet Public Health. 2018;3(7):e323–32. 10.1016/S2468-2667(18)30091-4. 29908859 10.1016/S2468-2667(18)30091-4 PMC6028743

[6] Buckinx F, Rolland Y, Reginster JY, Ricour C, Petermans J, Bruyère O. Burden of frailty in the elderly population: perspectives for a public health challenge. Arch Public Health. 2015;73(1):19. 10.1186/s13690-015-0068-x. 25866625 10.1186/s13690-015-0068-x PMC4392630

[7] Latham NK, Anderson CS, Lee A, et al. A randomized, controlled trial of quadriceps resistance exercise and vitamin D in frail older people: the Frailty interventions Trial in Elderly subjects (FITNESS). J Am Geriatr Soc. 2003;51(3):291–9. 10.1046/j.1532-5415.2003.51101.x. 12588571 10.1046/j.1532-5415.2003.51101.x

[8] Sun M, Wang L, Wang X, et al. Interaction between sleep quality and dietary inflammation on frailty: NHANES 2005–2008. Food Funct. 2023;14(2):1003–10. 10.1039/d2fo01832b. Published 2023 Jan 23. 36546877 10.1039/d2fo01832b

[9] Pond HM, Kehler S, Seaman K, Bouchard DR, Sénéchal M. Association between physical activity & sedentary time on frailty status in males and females living with diabetes mellitus: a cross-sectional analysis. Exp Gerontol. 2022;161:111741. 10.1016/j.exger.2022.111741. 35150826 10.1016/j.exger.2022.111741

[10] Song J, Lindquist LA, Chang RW, et al. Sedentary behavior as a risk factor for physical Frailty Independent of Moderate Activity: results from the Osteoarthritis Initiative. Am J Public Health. 2015;105(7):1439–45. 10.2105/AJPH.2014.302540. 25973826 10.2105/AJPH.2014.302540 PMC4463377

[11] Nakakubo S, Makizako H, Doi T, et al. Long and short sleep duration and physical Frailty in Community-Dwelling older adults. J Nutr Health Aging. 2018;22(9):1066–71. 10.1007/s12603-018-1116-3.30379304 10.1007/s12603-018-1116-3

[12] Rollo S, Antsygina O, Tremblay MS. The whole day matters: understanding 24-hour movement guideline adherence and relationships with health indicators across the lifespan. J Sport Health Sci. 2020;9(6):493–510. 10.1016/j.jshs.2020.07.004. 32711156 10.1016/j.jshs.2020.07.004 PMC7749249

[13] Liang W, Wang Y, Huang Q, et al. Adherence to 24-Hour Movement Guidelines among Chinese older adults: prevalence, correlates, and associations with Physical and Mental Health outcomes. JMIR Public Health Surveill. 2024;10:e46072. 10.2196/46072. 38869941 10.2196/46072 PMC11211711

[14] Duncan MJ, Holliday EG, Burton NW, Glozier N, Oftedal S. Prospective associations between joint categories of physical activity and insomnia symptoms with onset of poor mental health in a population-based cohort. J Sport Health Sci. 2023;12(3):295–303. 10.1016/j.jshs.2022.02.002. 35192936 10.1016/j.jshs.2022.02.002 PMC10199134

[15] McGregor DE, Palarea-Albaladejo J, Dall PM, Del Pozo Cruz B, Chastin SFM. Compositional analysis of the association between mortality and 24-hour movement behaviour from NHANES. Eur J Prev Cardiol. 2021;28(7):791–8. 10.1177/2047487319867783. 34247228 10.1177/2047487319867783

[16] Ross R, Chaput JP, Giangregorio LM, et al. Canadian 24-Hour Movement guidelines for adults aged 18–64 years and adults aged 65 years or older: an integration of physical activity, sedentary behaviour, and sleep. Appl Physiol Nutr Metab. 2020;45(10):S57–102. 10.1139/apnm-2020-0467. 33054332 10.1139/apnm-2020-0467

[17] Kong C, Chen A, Ludyga S, et al. Associations between meeting 24-hour movement guidelines and quality of life among children and adolescents with autism spectrum disorder. J Sport Health Sci. 2023;12(1):73–86. 10.1016/j.jshs.2022.08.003. 36029958 10.1016/j.jshs.2022.08.003 PMC9923433

[18] Rollo S, Lang JJ, Roberts KC, et al. Health associations with meeting the Canadian 24-hour movement guidelines for adults: results from the Canadian Health measures Survey. Health Rep. 2022;33(1):16–26. 10.25318/82-003-x202200100002-eng. 35050558 10.25318/82-003-x202200100002-eng

[19] Huang J, Li X, Li G, et al. Prevalence of meeting 24-hour movement guidelines and its associations with health indicators in people with disabilities: a systematic review and meta-analysis. Disabil Health J. 2024;17(3):101616. 10.1016/j.dhjo.2024.101616. 38514296 10.1016/j.dhjo.2024.101616

[20] Ferrari G, Alberico C, Drenowatz C et al. Prevalence and sociodemographic correlates of meeting the Canadian 24-hour movement guidelines among latin american adults: a multi-national cross-sectional study. BMC Public Health. 2022;22(1):217. Published 2022 Feb 3. 10.1186/s12889-022-12613-2 10.1186/s12889-022-12613-2 PMC8812134 35109819

[21] von Elm E, Altman DG, Egger M et al. The Strengthening the Reporting of Observational Studies in Epidemiology (STROBE) statement: guidelines for reporting observational studies [published correction appears in Ann Intern Med. 2008;148(2):168]. Ann Intern Med. 2007;147(8):573–577. 10.7326/0003-4819-147-8-200710160-00010 10.7326/0003-4819-147-8-200710160-00010 17938396

[22] Liang J, Huang S, Jiang N, et al. Association between Joint Physical Activity and Dietary Quality and Lower Risk of Depression symptoms in US Adults: cross-sectional NHANES Study. JMIR Public Health Surveill. 2023;9:e45776. 10.2196/45776. 37163324 10.2196/45776 PMC10209797

[23] Schuna JM Jr, Johnson WD, Tudor-Locke C. Adult self-reported and objectively monitored physical activity and sedentary behavior: NHANES 2005–2006. Int J Behav Nutr Phys Act. 2013;10:126. 10.1186/1479-5868-10-126. 24215625 10.1186/1479-5868-10-126 PMC3828579

[24] Cleland CL, Hunter RF, Kee F, Cupples ME, Sallis JF, Tully MA. Validity of the global physical activity questionnaire (GPAQ) in assessing levels and change in moderate-vigorous physical activity and sedentary behaviour. BMC Public Health. 2014;14:1255. 10.1186/1471-2458-14-1255. 25492375 10.1186/1471-2458-14-1255 PMC4295403

[25] Liang JH, Huang S, Pu YQ, et al. Whether weekend warrior activity and other leisure-time physical activity pattern reduce the risk of depression symptom in the representative adults? A population-based analysis of NHANES 2007–2020. J Affect Disord. 2023;340:329–39. 10.1016/j.jad.2023.07.113. 37543116 10.1016/j.jad.2023.07.113

[26] Mansoubi M, Pearson N, Clemes SA, et al. Energy expenditure during common sitting and standing tasks: examining the 1.5 MET definition of sedentary behaviour. BMC Public Health. 2015;15:516. 10.1186/s12889-015-1851-x. 26021449 10.1186/s12889-015-1851-x PMC4448542

[27] Hakeem FF, Bernabé E, Sabbah W. Association between Oral Health and Frailty among American older adults. J Am Med Dir Assoc. 2021;22(3):559–e5632. 10.1016/j.jamda.2020.07.023. 32859517 10.1016/j.jamda.2020.07.023

[28] Searle SD, Mitnitski A, Gahbauer EA, Gill TM, Rockwood K. A standard procedure for creating a frailty index. BMC Geriatr. 2008;8:24. 10.1186/1471-2318-8-24. 18826625 10.1186/1471-2318-8-24 PMC2573877

[29] Rockwood K, Mitnitski A. Frailty in relation to the accumulation of deficits. J Gerontol Biol Sci Med Sci. 2007;62(7):722–7. 10.1093/gerona/62.7.722. 10.1093/gerona/62.7.722 17634318

[30] Blodgett J, Theou O, Kirkland S, Andreou P, Rockwood K. Frailty in NHANES: comparing the frailty index and phenotype. Arch Gerontol Geriatr. 2015;60(3):464–70. 10.1016/j.archger.2015.01.016. 25697060 10.1016/j.archger.2015.01.016

[31] Liao J, Hu M, Imm K, et al. Association of daily sitting time and leisure-time physical activity with body fat among U.S. adults. J Sport Health Sci. 2024;13(2):195–203. 10.1016/j.jshs.2022.10.001. 36240998 10.1016/j.jshs.2022.10.001 PMC10980870

[32] Huo X, Jia S, Sun L, Yao Y, Liao H, Chen X. Association of dietary live microbe intake with frailty in US adults: evidence from NHANES. J Nutr Health Aging. 2024;28(3):100171. 10.1016/j.jnha.2024.100171.38423889 10.1016/j.jnha.2024.100171

[33] World Health Organization. Physical Activity. https://www.who.int/news-room/fact-sheets/detail/physical-activity. Accessed December 23, 2024.

[34] Zhao W, Hu P, Sun W, et al. Effect of physical activity on the risk of frailty: a systematic review and meta-analysis. PLoS ONE. 2022;17(12):e0278226. 10.1371/journal.pone.0278226. 36454790 10.1371/journal.pone.0278226 PMC9714708

[35] Blodgett J, Theou O, Kirkland S, Andreou P, Rockwood K. The association between sedentary behaviour, moderate-vigorous physical activity and frailty in NHANES cohorts. Maturitas. 2015;80(2):187–91. 10.1016/j.maturitas.2014.11.010. 25542406 10.1016/j.maturitas.2014.11.010

[36] Yu L, Guo Z, Long Q, et al. Modifiable lifestyle, sedentary behaviors and the risk of Frailty: a Univariate and Multivariate mendelian randomization study. Adv Biol (Weinh). 2024;8(5):e2400052. 10.1002/adbi.202400052. 38532244 10.1002/adbi.202400052

[37] Moreno-Tamayo K, Manrique-Espinoza B, Morales-Carmona E, Salinas-Rodríguez A. Sleep duration and incident frailty: the rural Frailty Study. BMC Geriatr. 2021;21(1):368. 10.1186/s12877-021-02272-0. Published 2021 Jun 16. 34134643 10.1186/s12877-021-02272-0 PMC8207661

[38] Salinas-Rodríguez A, Manrique-Espinoza B, Moreno-Tamayo K, Guerrero-Zúñiga S. Trajectories of sleep duration and quality and their association with mild cognitive impairment, frailty, and all-cause mortality. Sleep Health. 2024;10(2):240–8. 10.1016/j.sleh.2023.12.002. 38238122 10.1016/j.sleh.2023.12.002

[39] Kehler DS. The impact of sedentary and physical activity behaviour on frailty in middle-aged and older adults. Appl Physiol Nutr Metab. 2018;43(6):638. 10.1139/apnm-2018-0092. 29733701 10.1139/apnm-2018-0092

[40] Kehler DS, Theou O. The impact of physical activity and sedentary behaviors on frailty levels. Mech Ageing Dev. 2019;180:29–41. 10.1016/j.mad.2019.03.004. 30926562 10.1016/j.mad.2019.03.004

[41] Zhou Y, Zhu J, Huang Y, et al. Physical activity, sedentary behavior, and the risk of frailty and falling: a mendelian randomization study. Scand J Med Sci Sports. 2024;34(2):e14582. 10.1111/sms.14582. 38349064 10.1111/sms.14582

[42] Gordon EH, Hubbard RE. Frailty: understanding the difference between age and ageing. Age Ageing. 2022;51(8):afac185. 10.1093/ageing/afac185. 35973066 10.1093/ageing/afac185

[43] Li C, Li N, Huang H, Li Y, Zhuang Y. Causal associations between leisure sedentary behaviors and sleep status with frailty: insight from mendelian randomization study. BMC Geriatr. 2024;24(1):168. 10.1186/s12877-024-04758-z. Published 2024 Feb 17. 38368347 10.1186/s12877-024-04758-z PMC10874533

[44] Kehler DS, Clara I, Hiebert B, et al. Sex-differences in relation to the association between patterns of physical activity and sedentary behavior with frailty. Arch Gerontol Geriatr. 2020;87:103972. 10.1016/j.archger.2019.103972. 31739110 10.1016/j.archger.2019.103972

[45] Mong JA, Cusmano DM. Sex differences in sleep: impact of biological sex and sex steroids. Philos Trans R Soc Lond B Biol Sci. 2016;371(1688):20150110. 10.1098/rstb.2015.0110. 26833831 10.1098/rstb.2015.0110 PMC4785896

[46] Zhou Y, Li Z, Li J, et al. Sex difference in the Association between Sedentary Behavior and Sleep Quality: a longitudinal study among older adults in Rural China. J Am Med Dir Assoc. 2023;24(10):1520–e15262. 10.1016/j.jamda.2023.03.022. 37105235 10.1016/j.jamda.2023.03.022

[47] Memon AR, Gupta CC, Crowther ME, Ferguson SA, Tuckwell GA, Vincent GE. Sleep and physical activity in university students: a systematic review and meta-analysis. Sleep Med Rev. 2021;58:101482. 10.1016/j.smrv.2021.101482. 33864990 10.1016/j.smrv.2021.101482

[48] Pesonen AK, Kahn M, Kuula L et al. Sleep and physical activity - the dynamics of bi-directional influences over a fortnight. BMC Public Health. 2022;22(1):1160. Published 2022 Jun 10. 10.1186/s12889-022-13586-y 10.1186/s12889-022-13586-y PMC9185923 35681198

[49] Muscedere J, Waters B, Varambally A, et al. The impact of frailty on intensive care unit outcomes: a systematic review and meta-analysis. Intensive Care Med. 2017;43(8):1105–22. 10.1007/s00134-017-4867-0. 28676896 10.1007/s00134-017-4867-0 PMC5501903

[50] Chowdhury R, Peel NM, Krosch M, Hubbard RE. Frailty and chronic kidney disease: a systematic review. Arch Gerontol Geriatr. 2017;68:135–42. 10.1016/j.archger.2016.10.007. 27810661 10.1016/j.archger.2016.10.007

[51] Yee N, Locke ER, Pike KC, et al. Frailty in Chronic Obstructive Pulmonary Disease and Risk of exacerbations and hospitalizations. Int J Chron Obstruct Pulmon Dis. 2020;15:1967–76. 10.2147/COPD.S245505. Published 2020 Aug 11. 32848382 10.2147/COPD.S245505 PMC7429100

[52] Stewart R. Cardiovascular Disease and Frailty: what are the mechanistic links? Clin Chem. 2019;65(1):80–6. 10.1373/clinchem.2018.287318. 30504259 10.1373/clinchem.2018.287318

[53] Vetrano DL, Palmer K, Marengoni A, et al. Frailty and Multimorbidity: a systematic review and Meta-analysis. J Gerontol Biol Sci Med Sci. 2019;74(5):659–66. 10.1093/gerona/gly110. 10.1093/gerona/gly110 29726918

[54] Kastelic K, Pedišić Ž, Lipovac D, Kastelic N, Chen ST, Šarabon N. Associations of meeting 24-h movement guidelines with stress and self-rated health among adults: is meeting more guidelines associated with greater benefits? BMC Public Health. 2021;21(1):929. 10.1186/s12889-021-10979-3. 34001090 10.1186/s12889-021-10979-3 PMC8127279

[55] Ferrari G, Cristi-Montero C, Drenowatz C et al. Meeting 24-h movement guidelines and markers of adiposity in adults from eight Latin America countries: the ELANS study. Sci Rep. 2022;12(1):11382. Published 2022 Jul 5. 10.1038/s41598-022-15504-z 10.1038/s41598-022-15504-z PMC9256603 35790777

[56] Shin SW, Choi Y, Kang YH, Kim J. Associations of meeting 24-h movement guidelines and metabolic syndrome in Korean adults during the COVID-19 pandemic. Public Health. 2024;227:187–93. 10.1016/j.puhe.2023.12.020. 38237314 10.1016/j.puhe.2023.12.020

[57] Dooley EE, Palta P, Wolff-Hughes DL, et al. Higher 24-h Total Movement Activity Percentile is Associated with Better Cognitive performance in U.S. older adults. Med Sci Sports Exerc. 2022;54(8):1317–25. 10.1249/MSS.0000000000002927. 35389933 10.1249/MSS.0000000000002927 PMC9288525

[58] García-Hermoso A, Ezzatvar Y, Ramírez-Vélez R, López-Gil JF, Izquierdo M. Trajectories of 24-h movement guidelines from middle adolescence to adulthood on depression and suicidal ideation: a 22-year follow-up study. Int J Behav Nutr Phys Act. 2022;19(1):135. Published 2022 Oct 23. 10.1186/s12966-022-01367-0 10.1186/s12966-022-01367-0 PMC9590171 36274150

[59] Yang Z, Li X, Song W, Zhang Y. Associations between meeting 24-h movement guidelines and Sarcopenia risk among adults aged ≥ 55 years in five low- and middle-income countries. Complement Ther Clin Pract Published Online July. 2024;24. 10.1016/j.ctcp.2024.101887. 10.1016/j.ctcp.2024.101887 39084129

