# Supplementary material for: Is meeting 24-hour movement guidelines associated with a lower risk of frailty among adults?
Source: Int J Behav Nutr Phys Act. 2025 Feb 21;22:21. doi: 10.1186/s12966-025-01722-x (PMC11846395; doi:10.1186/s12966-025-01722-x)
Supplement: Supplementary file 1 — Supplementary Material 1: Supplementary Fig. 1 Associations of meeting 24-h movement guidelines with frailty (the cut of point = 0.25). Supplementary Table 1 Variables in the 49-item frailty index and their respective scorings. Supplementary Table 2 Baseline characteristics of study participants stratified by meeting 24-h movement guidelines. Supplementary Table 3 Baseline characteristics of study participants stratified by the number of guidelines met. Supplementary Table 4 Baseline characteristics of study participants stratified by age. Supplementary Table 5 Baseline characteristics of study participants stratified by gender. Supplementary Table 6 Baseline characteristics of study participants stratified by race/ethnicity. Supplementary Table 7 Associations of meeting 24-h movement guidelines with frailty index (continuous variable). Supplementary Table 8 Further adjustments in sensitivity analyses for associations of meeting 24-h movement guidelines with frailty [file 12966_2025_1722_MOESM1_ESM.docx]

**Supplementary materials**

**Is meeting 24-h movement guidelines associated with a lower risk of frailty among adults?**

Yuhang Liu^1^, Siyao Gao^2*^, Zhigang Dou^3^, Zhen Chen^2^, Jialing Tang^2^

* Correspondence: gaosiyao@csu.edu.cn

^1^ School of Physical Education and Sports, Central China Normal University, Wuhan 430079, P. R. China.

^2^ Department of Physical Education, Central South University, Changsha, 410083, P. R. China.

^3^ Henan Experimental High School, Zhengzhou, 450002, P. R. China.

| **Content** | |
| --- | --- |
| **Supplemental figures and tables** | **Page** |
| **Fig. 1** Associations of meeting 24-h movement guidelines with frailty (the cut of point = 0.25). | 3 |
| **Table 1** Variables in the 49-Item Frailty Index and Their Respective Scorings. | 4-5 |
| **Table 2** Baseline characteristics of study participants stratified by meeting 24-h movement guidelines. | 6-8 |
| **Table 3** Baseline characteristics of study participants stratified by the number of guidelines met. | 9-10 |
| **Table 4** Baseline characteristics of study participants stratified by age. | 11-12 |
| **Table 5** Baseline characteristics of study participants stratified by gender. | 13-14 |
| **Table 6** Baseline characteristics of study participants stratified by race/ethnicity. | 15-16 |
| **Table 7** Associations of meeting 24-h movement guidelines with frailty index. | 17 |
| **Table 8** Further adjustments in sensitivity analyses for associations of meeting 24-h movement guidelines with frailty. | 18 |

**Supplementary Fig. 1 Associations of meeting 24-h movement guidelines with frailty (the cut of point = 0.25).**

The results of COR (95% CI), AOR (95% CI), and *P*-value shown in bold were statistically significant. Model 1 was adjusted for age, gender, race/ethnicity. Model 2 was additionally adjusted for education level, marital status, PIR, BMI, smoking, alcohol consumption and total energy intake. AOR, Adjusted odds ratio; BMI, Body mass index; CI, Confidence interval; COR, Crude odds ratio; MVPA, Moderate-to-vigorous physical activity; PIR, Poverty income ratio; SB, Sedentary behavior.

| **Supplementary Table 1 Variables in the 49-Item Frailty Index and Their Respective Scorings.** | | |
| --- | --- | --- |
| **Variable** | **Scoring** | |
| **Cognition** |  | |
| 1. Experience confusion/memory problems | Yes = 1, No = 0 | |
| **Dependence** |  | |
| 2. Managing money | Difficulty = 1, No Difficulty = 0 | |
| 3. Stooping, crouching, kneeling | Difficulty = 1, No Difficulty = 0 | |
| 4. Lifting or carrying | Difficulty = 1, No Difficulty = 0 | |
| 5. House chore | Difficulty = 1, No Difficulty = 0 | |
| 6. Preparing meals | Difficulty = 1, No Difficulty = 0 | |
| 7. Standing up from armless chair | Difficulty = 1, No Difficulty = 0 | |
| 8. Getting in and out of bed difficulty | Difficulty = 1, No Difficulty = 0 | |
| 9. Using fork, knife, drinking from cup | Difficulty = 1, No Difficulty = 0 | |
| 10. Dressing yourself | Difficulty = 1, No Difficulty = 0 | |
| 11. Standing for long periods difficulty | Difficulty = 1, No Difficulty = 0 | |
| 12. Grasp/holding small objects | Difficulty = 1, No Difficulty = 0 | |
| 13. Attending social event | Difficulty = 1, No Difficulty = 0 | |
| 14. Push or pull large objects | Difficulty = 1, No Difficulty = 0 | |
| 15. Walking for a quarter mile difficulty | Difficulty = 1, No Difficulty = 0 | |
| 16. Walking up 10 steps difficulty | Difficulty = 1, No Difficulty = 0 | |
| **Depressive Symptoms** |  | |
| 17. Have little interest in doing things | Nearly every day = 1, More than half the days = 0.66, Several days = 0.33, Not at all = 0 | |
| 18. Feeling down, depressed, or hopeless | Nearly every day = 1, More than half the days = 0.66, Several days = 0.33, Not at all = 0 | |
| 19. Trouble sleeping or sleeping too much | Nearly every day = 1, More than half the days = 0.66, Several days = 0.33, Not at all = 0 | |
| 20. Feeling tired or having little energy | Nearly every day = 1, More than half the days = 0.66, Several days = 0.33, Not at all = 0 | |
| 21. Poor appetite or overeating | Nearly every day = 1, More than half the days = 0.66, Several days = 0.33, Not at all = 0 | |
| 22. Feeling bad about yourself | Nearly every day = 1, More than half the days = 0.66, Several days = 0.33, Not at all = 0 | |
| 23. Trouble concentrating on things | Nearly every day = 1, More than half the days = 0.66, Several days = 0.33, Not at all = 0 | |
| **Comorbidities** |  | |
| 24. Arthritis | Yes = 1, Suspect = 0.5 No = 0 | |
| 25. Thyroid problems | Yes = 1, Suspect = 0.5 No = 0 | |
| 26. Chronic bronchitis | Yes = 1, Suspect = 0.5 No = 0 | |
| 27. Cancer | Yes = 1, Suspect = 0.5 No = 0 | |
| 28. Congestive heart failure | Yes = 1, Suspect = 0.5 No = 0 | |
| 29. Coronary heart disease | Yes = 1, Suspect = 0.5 No = 0 | |
| 30. Angina | Yes = 1, Suspect = 0.5 No = 0 | |
| 31. Heart attack | Yes = 1, Suspect = 0.5 No = 0 | |
| 32. Stroke | Yes = 1, Suspect = 0.5 No = 0 | |
| 33. Blood pressure | Yes = 1, Suspect = 0.5 No = 0 | |
| 34. Diabetes | Yes = 1, Suspect = 0.5 No = 0 | |
| 35. Weak/failing kidneys | Yes = 1, Suspect = 0.5 No = 0 | |
| 36. Urinary Leakage | Yes = 1, Suspect = 0.5 No = 0 | |
| **Hospital Utilization and Access to Care** |  | |
| 37. Self-rated health | Fair, poor = 1, Excellent, Very good, good = 0 | |
| 38. Health now compared with 1 year ago | Worse = 1, About the same, better = 0 | |
| 39. Overnight hospital patient in past year | Yes =1, No =0 | |
| 40. Frequency of health care use during past year | None = 0, 1-5 = 0,5, More than 5 = 1 | |
| 41. Number of prescribed medications | None = 0, 1-5 = 0,5, More than 5 = 1 | |
| **Physical Performance and Anthropometry** |  | |
| 42.Body mass index (kg/m^2^) | < 18.5, ≥ 30 = 1  ≥ 25, < 30 = 0.5  ≥ 18.5,＜ 25 = 0 | |
| 43. Handgrip strength | MALE: For BMI ≤ 24, GS ≤ 29 For BMI 24.1-28, GS ≤ 30 For BMI > 28, GS ≤ 32 = 1 | FEMALE: For BMI ≤ 23, GS ≤ 17 For BMI 23.1-26, GS ≤ 17.3 For BMI 26.1-29, GS ≤ 18 For BMI > 29, GS ≤ 21 = 1 |
| **Laboratory Values** |  | |
| 44. Glycohemoglobin (%) | 0%-5.7% = 0, >5.7% = 1 | |
| 45. Red blood cell count (million cells/mL) | M: 4.7-6.1 = 0, Other = 1 | F: 4.2-5.4 = 0, Other = 1 |
| 46. Hemoglobin (g/dL) | M: 13.5-18 =0, Other = 1 | F:12-16 = 0, Other = 1 |
| 47. Red cell distribution width (%) | 11.6-14.6 = 0, Other = 1 | |
| 48. Lymphocyte percent (%) | 2040 = 0, Other= 1 | |
| 49. Segmented neutrophils percent (%) | 40-80 = 0, Other = 1 | |
| **Abbreviation:** BMI, Body mass index; GS, grip strength.  **References**:  Hakeem FF, Bernabé E, Sabbah W. Association Between Oral Health and Frailty Among American Older Adults. J Am Med Dir Assoc. 2021;22(3):559-563.e2. doi:10.1016/j.jamda.2020.07.023 | | |

| **Supplementary Table 2 Baseline characteristics of study participants stratified by meeting 24-h movement guidelines.** | | | | | | | | | | | |
| --- | --- | --- | --- | --- | --- | --- | --- | --- | --- | --- | --- |
|  | **Total participants** | **None** | **Meeting individual guidelines** | | |  | **Meeting specific guideline combinations** | | | **MVPA + SB + Sleep** | ***P*-value^a^** |
|  |  |  | **MVPA** | **SB** | **Sleep** |  | **SB + Sleep** | **MVPA + Sleep** | **MVPA + SB** |  |  |
| **Age** | 50.25 ± 0.31 | 51.22 ± 0.66 | 48.31 ± 1.02 | 50.40 ± 0.55 | 52.87± 0.60 |  | 53.36 ± 0.60 | 49.86 ± 0.81 | 47.83 ± 0.63 | 49.66 ± 0.58 | **< 0.001** |
| 18-39 | 1,090 (21.9) | 88 (18.2) | 94 (25.4) | 98 (17.5) | 65 (13.7) |  | 87 (13.7) | 116 (23.2) | 253 (28.8) | 289 (25.7) | **< 0.001** |
| 40-59 | 2,170 (41.6) | 228 (48.8) | 153 (43.2) | 319 (54.0) | 217 (43.9) |  | 281 (40.7) | 166 (38.8) | 388 (42.2) | 418 (34.2) |  |
| 60- | 2,466 (36.5) | 208 (33.0) | 139 (31.4) | 301 (28.5) | 252 (42.5) |  | 394 (45.5) | 213 (38.0) | 394 (28.9) | 565 (40.1) |  |
| **Education attainment** |  |  |  |  |  |  |  |  |  |  |  |
| Less than high school | 569 (4.8) | 46 (5.5) | 11 (2.2) | 104 (8.9) | 39 (4.4) |  | 113 (7.3) | 12 (0.8) | 98 (4.3) | 146 (5.0) | **< 0.001** |
| High school | 2,372 (38.1) | 222 (41.1) | 158 (34.7) | 345 (47.8) | 196 (31.6) |  | 366 (45.4) | 137 (22.5) | 483 (46.9) | 465 (34.8) |  |
| More than high school | 2,785 (57.1) | 256 (53.4) | 217 (63.1) | 269 (43.3) | 299 (64.0) |  | 283 (47.2) | 346 (76.7) | 454 (48.8) | 661 (60.2) |  |
| **PIR** | 2.75 ± 0.05 | 2.51 ± 0.12 | 2.79 ± 0.13 | 1.95 ± 0.09 | 3.10 ± 0.13 |  | 2.57 ± 0.09 | 3.54 ± 0.10 | 2.44 ± 0.08 | 2.89 ± 0.07 | **< 0.001** |
| <1.3 | 2,394 (29.7) | 243 (35.7) | 157 (30.8) | 372 (43.5) | 188 (22.8) |  | 344 (32.6) | 128 (15.1) | 454 (34.2) | 508 (27.4) | **< 0.001** |
| 1.3-3.5 | 1,871 (32.2) | 154 (30.3) | 122 (29.8) | 250 (39.3) | 169 (29.9) |  | 254 (33.5) | 144 (25.3) | 362 (36.0) | 416 (31.8) |  |
| >3.5 | 1,461 (38.1) | 127 (34.0) | 107 (39.4) | 96 (17.2) | 177 (47.3) |  | 164 (33.8) | 223 (59.6) | 219 (29.9) | 348 (40.8) |  |
| **Gender** |  |  |  |  |  |  |  |  |  |  |  |
| Female | 2,956 (52.4) | 279 (55.6) | 181 (46.4) | 427 (58.8) | 326 (61.1) |  | 459 (63.2) | 234 (45.3) | 459 (47.1) | 591 (48.5) | **< 0.001** |
| Male | 2,770 (47.6) | 245 (44.4) | 205 (53.6) | 291 (41.2) | 208 (38.9) |  | 303 (36.8) | 261 (54.7) | 576 (52.9) | 681 (51.5) |  |
| **Race/ethnicity** |  |  |  |  |  |  |  |  |  |  |  |
| Non-Hispanic White | 2,435 (71.4) | 242 (70.1) | 172 (72.2) | 242 (58.8) | 257 (75.6) |  | 271 (67.2) | 281 (83.6) | 416 (68.7) | 554 (72.9) | **< 0.001** |
| Non-Hispanic Black | 1,382 (11.3) | 149 (14.8) | 126 (16.6) | 208 (17.6) | 131 (10.0) |  | 167 (10.9) | 87 (6.0) | 282 (13.5) | 232 (7.9) |  |
| Mexican American | 778 (5.9) | 40 (4.5) | 30 (3.5) | 124 (9.5) | 58 (4.4) |  | 154 (8.4) | 37 (3.0) | 131 (5.7) | 204 (6.7) |  |
| Other Races | 1,131 (11.3) | 93 (10.6) | 58 (7.7) | 144 (14.1) | 88 (10.0) |  | 170 (13.5) | 90 (7.4) | 206 (12.1) | 282 (12.6) |  |
| **BMI** | 30.60 ± 0.16 | 34.41 ± 0.65 | 30.59 ± 0.50 | 30.41 ± 0.44 | 33.40 ± 0.41 |  | 30.29 ± 0.35 | 30.02 ± 0.44 | 29.94 ± 0.31 | 29.03 ± 0.30 | **< 0.001** |
| Underweight (<18.5) | 99 (1.9) | 12 (2.8) | 9 (1.9) | 14 (3.1) | 3 (0.3) |  | 15 (1.6) | 6 (1.1) | 20 (2.2) | 20 (2.0) | **< 0.001** |
| Normal (18.5-25) | 1,207 (22.0) | 70 (11.2) | 79 (24.0) | 153 (21.6) | 101 (14.8) |  | 156 (21.4) | 103 (22.6) | 234 (22.7) | 311 (27.9) |  |
| Overweight (25-30) | 1,701 (29.5) | 108 (21.3) | 97 (23.9) | 215 (32.0) | 130 (24.3) |  | 228 (28.5) | 170 (35.7) | 326 (31.5) | 427 (31.4) |  |
| Obese (≥30) | 2,719 (46.6) | 334 (64.7) | 201 (50.2) | 336 (43.3) | 300 (60.6) |  | 363 (48.5) | 216 (40.7) | 455 (43.6) | 514 (38.7) |  |
| **Marial status** |  |  |  |  |  |  |  |  |  |  |  |
| Married/Living with Partner | 3,231 (60.9) | 277 (56.5) | 182 (43.5) | 376 (53.3) | 299 (62.9) |  | 466 (66.3) | 290 (65.6) | 574 (58.3) | 767 (66.5) | **< 0.001** |
| Never married | 969 (16.9) | 75 (14.6) | 91 (25.8) | 109 (17.4) | 68 (12.3) |  | 102 (12.7) | 95 (17.7) | 197 (18.8) | 232 (17.0) |  |
| Widowed/Divorced/Separated | 1,526 (22.2) | 172 (28.8) | 113 (30.7) | 233 (29.2) | 167 (24.7) |  | 194 (21.0) | 110 (16.7) | 264 (22.9) | 273 (16.6) |  |
| **Smoke status** |  |  |  |  |  |  |  |  |  |  |  |
| Never | 2,570 (45.8) | 213 (43.2) | 163 (45.2) | 316 (37.8) | 246 (49.0) |  | 391 (51.0) | 248 (55.5) | 446 (43.6) | 547 (42.9) | **< 0.001** |
| Former | 1,464 (26.5) | 137 (23.0) | 79 (22.9) | 159 (24.2) | 146 (29.2) |  | 187 (23.8) | 139 (26.3) | 251 (24.5) | 366 (31.3) |  |
| Current | 1,692 (27.7) | 174 (33.9) | 144 (31.9) | 243 (38.0) | 142 (21.8) |  | 184 (25.2) | 108 (18.2) | 338 (32.0) | 359 (25.8) |  |
| **Alcohol consumption** |  |  |  |  |  |  |  |  |  |  |  |
| Never | 746 (10.2) | 57 (9.0) | 35 (6.7) | 118 (12.9) | 66 (9.5) |  | 134 (13.3) | 53 (10.5) | 119 (9.5) | 164 (9.7) | **< 0.001** |
| Current | 3,876 (73.5) | 334 (66.6) | 283 (80.3) | 432 (63.8) | 361 (73.6) |  | 451 (65.8) | 368 (78.5) | 733 (75.5) | 914 (77.6) |  |
| Former | 1,104 (16.3) | 133 (24.4) | 68 (13.0) | 168 (23.3) | 107 (16.8) |  | 177 (20.9) | 74 (10.9) | 183 (15.0) | 194 (12.7) |  |
| **Hypertension** |  |  |  |  |  |  |  |  |  |  |  |
| No | 2,612 (50.0) | 181 (32.1) | 165 (49.0) | 296 (47.5) | 226 (43.3) |  | 322 (42.4) | 261 (57.9) | 472 (52.3) | 689 (59.0) | **< 0.001** |
| Yes | 3,114 (50.0) | 343 (67.9) | 221 (51.0) | 422 (52.5) | 308 (56.7) |  | 440 (57.6) | 234 (42.1) | 563 (47.7) | 583 (41.0) |  |
| **DM** |  |  |  |  |  |  |  |  |  |  |  |
| No | 3,780 (71.6) | 301 (59.6) | 263 (72.2) | 435 (66.3) | 309 (60.4) |  | 479 (68.0) | 367 (79.1) | 718 (75.9) | 908 (77.8) | **< 0.001** |
| Yes | 1,946 (28.4) | 223 (40.4) | 123 (27.8) | 283 (33.7) | 225 (39.6) |  | 283 (32.0) | 128 (20.9) | 317 (24.1) | 364 (22.2) |  |
| **CVDs** |  |  |  |  |  |  |  |  |  |  |  |
| No | 4,894 (87.7) | 416 (80.4) | 328 (87.4) | 584 (84.0) | 447 (86.3) |  | 654 (87.5) | 441 (91.2) | 898 (89.0) | 1126 (89.8) | **< 0.001** |
| Yes | 832 (12.3) | 108 (19.6) | 58 (12.6) | 134 (16.0) | 87 (13.7) |  | 108 (12.5) | 54 (8.8) | 137 (11.0) | 146 (10.2) |  |
| **SB time (Minutes/per day)** | 381.59 ± 4.11 | 624.58 ± 9.21 | 596.22 ± 8.74 | 245.72 ± 6.52 | 606.84 ± 6.33 |  | 258.70 ± 5.26 | 584.34 ± 6.35 | 242.25 ± 4.28 | 249.67±4.07 | **< 0.001** |
| **Sleep time** | 7.06 ± 0.03 | 6.28 ± 0.12 | 6.35 ± 0.17 | 6.11 ± 0.11 | 7.74 ± 0.04 |  | 7.79 ± 0.04 | 7.66 ± 0.04 | 6.05 ± 0.10 | 7.70±0.03 | **< 0.001** |
| **Total MVPA (Minutes/per week)** | 1,175.71 ± 41.54 | 77.75 ± 3.00 | 1,105.02 ± 102.41 | 77.43 ± 3.05 | 72.41 ± 3.39 |  | 74.40 ± 3.52 | 715.67 ± 43.89 | 1,812.75 ± 97.78 | 1,589.42 ± 75.13 | **< 0.001** |
| **Total energy intake (kcal)** | 2,147.56 ± 16.93 | 2,060.96 ± 64.77 | 2,352.39 ± 87.45 | 2,005.09 ± 58.05 | 2,058.73 ± 42.23 |  | 2,033.89 ± 45.72 | 2,203.21 ± 55.82 | 2,190.33 ± 49.69 | 2,210.14 ± 34.00 | **< 0.001** |
| **Year cycle** |  |  |  |  |  |  |  |  |  |  |  |
| 2007-2008 | 1,014 (15.0) | 85 (14.8) | 47 (10.6) | 167 (20.9) | 71 (12.5) |  | 163 (17.9) | 51 (9.4) | 203 (17.8) | 227 (14.5) | **< 0.001** |
| 2009-2010 | 1,018 (14.7) | 88 (13.9) | 58 (12.4) | 158 (18.7) | 84 (14.8) |  | 158 (18.1) | 57 (10.6) | 210 (17.1) | 205 (12.7) |  |
| 2011-2012 | 933 (17.7) | 96 (17.8) | 68 (17.0) | 125 (19.4) | 76 (13.6) |  | 109 (18.0) | 89 (19.0) | 172 (18.6) | 198 (17.3) |  |
| 2013-2014 | 985 (18.1) | 130 (25.7) | 99 (23.2) | 106 (18.2) | 139 (25.8) |  | 81 (12.0) | 122 (22.7) | 131 (13.7) | 177 (14.4) |  |
| 2015-2016 | 971 (18.5) | 74 (16.9) | 61 (18.2) | 91 (12.3) | 107 (25.0) |  | 148 (16.1) | 100 (21.7) | 160 (14.7) | 230 (21.1) |  |
| 2017-2018 | 805 (16.1) | 51 (10.9) | 53 (18.7) | 71 (10.6) | 57 (8.3) |  | 103 (17.9) | 76 (16.5) | 159 (18.1) | 235 (20.0) |  |
| **Frailty** |  |  |  |  |  |  |  |  |  |  |  |
| No | 3,639 (68.6) | 229 (50.5) | 236 (66.1) | 320 (47.3) | 300 (60.0) |  | 493 (66.7) | 396 (83.5) | 681 (70.0) | 984 (80.2) | **< 0.001** |
| Yes | 2,087 (31.4) | 295 (49.5) | 150 (33.9) | 398 (52.7) | 234 (40.0) |  | 269 (33.3) | 99 (16.5) | 354 (30.0) | 288 (19.8) |  |
| Footnotes: Continuous variables are presented as mean ± SE, and categorical variables are presented as n (weighted %). ^a^ *P*-values were assessed by one way ANOVA (continuous variables) or by Chi-square test (categorical variables). *P*-values shown in bold were statistically significant. Abbreviations: BMI, Body mass index; CVDs, Cardiovascular diseases; DM, Diabetes mellitus; MVPA, Moderate-to-vigorous physical activity; PIR, Poverty income ratio; SB, Sedentary behavior; SE, Standard error. | | | | | | | | | | | |

| **Supplementary Table 3 Baseline characteristics of study participants stratified by the number of guidelines met.** | | | | | | |
| --- | --- | --- | --- | --- | --- | --- |
|  | **Total participants** | **Number of guidelines met** | | | | ***P*-value^a^** |
|  |  | **0** | **1** | **2** | **3** |  |
| **Age** | 50.25 ± 0.31 | 51.22 ± 0.66 | 50.80 ± 0.43 | 50.04 ± 0.43 | 49.66 ± 0.58 | 0.2 |
| 18-39 | 1,090 (21.9) | 88 (18.2) | 257 (18.1) | 456 (22.8) | 289 (25.7) | **< 0.001** |
| 40-59 | 2,170 (41.6) | 228 (48.8) | 689 (47.2) | 835 (40.8) | 418 (34.2) |  |
| 60- | 2,466 (36.5) | 208 (33.0) | 692 (34.7) | 1,001 (36.4) | 565 (40.1) |  |
| **Education attainment** |  |  |  |  |  |  |
| Less than high school | 569 (4.8) | 46 (5.5) | 154 (5.4) | 223 (4.2) | 146 (5.0) | 0.1 |
| High school | 2,372 (38.1) | 222 (41.1) | 699 (38.0) | 986 (39.5) | 465 (34.8) |  |
| More than high school | 2,785 (57.1) | 256 (53.4) | 785 (56.6) | 1,083 (56.3) | 661 (60.2) |  |
| **PIR** | 2.75 ± 0.05 | 2.51 ± 0.12 | 2.62 ± 0.08 | 2.80 ± 0.07 | 2.89 ± 0.07 | **<0.05** |
| <1.3 | 2,394 (29.7) | 243 (35.7) | 717 (32.1) | 926 (28.2) | 508 (27.4) | 0.1 |
| 1.3-3.5 | 1,871 (32.2) | 154 (30.3) | 541 (33.1) | 760 (32.2) | 416 (31.8) |  |
| >3.5 | 1,461 (38.1) | 127 (34.0) | 380 (34.7) | 606 (39.6) | 348 (40.8) |  |
| **Gender** |  |  |  |  |  |  |
| Female | 2,956 (52.4) | 279 (55.6) | 934 (56.4) | 1,152 (51.3) | 591 (48.5) | **<0.05** |
| Male | 2,770 (47.6) | 245 (44.4) | 704 (43.6) | 1,140 (48.7) | 681 (51.5) |  |
| **Race/ethnicity** |  |  |  |  |  |  |
| Non-Hispanic White | 2,435 (71.4) | 242 (70.1) | 671 (68.9) | 968 (72.6) | 554 (72.9) | **< 0.001** |
| Non-Hispanic Black | 1,382 (11.3) | 149 (14.8) | 465 (14.4) | 536 (10.6) | 232 (7.9) |  |
| Mexican American | 778 (5.9) | 40 (4.5) | 212 (5.9) | 322 (5.7) | 204 (6.7) |  |
| Other Races | 1,131 (11.3) | 93 (10.6) | 290 (10.8) | 466 (11.1) | 282 (12.6) |  |
| **BMI** | 30.60 ± 0.16 | 34.41 ± 0.65 | 31.61 ± 0.24 | 30.07 ± 0.23 | 29.03 ± 0.30 | **< 0.001** |
| Underweight (<18.5) | 99 (1.9) | 12 (2.8) | 26 (1.7) | 41 (1.7) | 20 (2.0) | **< 0.001** |
| Normal (18.5-25) | 1,207 (22.0) | 70 (11.2) | 333 (19.6) | 493 (22.3) | 311 (27.9) |  |
| Overweight (25-30) | 1,701 (29.5) | 108 (21.3) | 442 (26.9) | 724 (31.8) | 427 (31.4) |  |
| Obese (≥30) | 2,719 (46.6) | 334 (64.7) | 837 (51.8) | 1,034 (44.2) | 514 (38.7) |  |
| **Marial status** |  |  |  |  |  |  |
| Married/Living with Partner | 3,231 (60.9) | 277 (56.5) | 857 (54.4) | 1,330 (62.7) | 767 (66.5) | **< 0.001** |
| Never married | 969 (16.9) | 75 (14.6) | 268 (17.7) | 394 (16.7) | 232 (17.0) |  |
| Widowed/Divorced/Separated | 1,526 (22.2) | 172 (28.8) | 513 (27.9) | 568 (20.6) | 273 (16.6) |  |
| **Smoke status** |  |  |  |  |  |  |
| Never | 2,570 (45.8) | 213 (43.2) | 725 (44.1) | 1,085 (49.2) | 547 (42.9) | **< 0.05** |
| Former | 1,464 (26.5) | 137 (23.0) | 384 (25.8) | 577 (24.8) | 366 (31.3) |  |
| Current | 1,692 (27.7) | 174 (33.9) | 529 (30.1) | 630 (26.0) | 359 (25.8) |  |
| **Alcohol consumption** |  |  |  |  |  |  |
| Never | 746 (10.2) | 57 (9.0) | 219 (10.0) | 306 (10.9) | 164 (9.7) | **< 0.001** |
| Current | 1,104 (16.3) | 133 (24.4) | 343 (18.1) | 434 (15.6) | 194 (12.7) |  |
| Former | 3,876 (73.5) | 334 (66.6) | 1,076 (72.0) | 1,552 (73.5) | 914 (77.6) |  |
| **Hypertension** |  |  |  |  |  |  |
| No | 2,612 (50.0) | 181 (32.1) | 687 (46.3) | 1,055 (51.0) | 689 (59.0) | **< 0.001** |
| Yes | 3,114 (50.0) | 343 (67.9) | 951 (53.7) | 1,237 (49.0) | 583 (41.0) |  |
| **DM** |  |  |  |  |  |  |
| No | 3,780 (71.6) | 301 (59.6) | 1,007 (65.5) | 1,564 (74.5) | 908 (77.8) | **< 0.001** |
| Yes | 1,946 (28.4) | 223 (40.4) | 631 (34.5) | 728 (25.5) | 364 (22.2) |  |
| **CVDs** |  |  |  |  |  |  |
| No | 4,894 (87.7) | 416 (80.4) | 1,359 (85.8) | 1,993 (89.2) | 1126 (89.8) | **< 0.001** |
| Yes | 832 (12.3) | 108 (19.6) | 279 (14.2) | 299 (10.8) | 146 (10.2) |  |
| **SB time (Minutes/per day)** | 381.59 ± 4.11 | 624.58 ± 9.21 | 478.69 ± 6.89 | 345.18 ± 7.17 | 249.67 ± 4.07 | **< 0.001** |
| **Sleep time** | 7.06 ± 0.03 | 6.28 ± 0.12 | 6.80 ± 0.07 | 7.02 ± 0.05 | 7.70 ± 0.03 | **< 0.001** |
| **Total MVPA (Minutes/per week)** | 1,175.71 ± 41.54 | 77.75 ± 3.00 | 610.93 ± 65.16 | 1,192.70 ± 58.17 | 1589.42 ± 75.13 | **< 0.001** |
| **Total energy intake (kcal)** | 2,147.56 ± 16.93 | 2060.96 ± 64.77 | 2118.24 ± 35.99 | 2,148.11 ± 30.13 | 2210.14 ± 34.00 | 0.1 |
| **Year cycle** |  |  |  |  |  |  |
| 2007-2008 | 1,014 (15.0) | 85 (14.8) | 285 (14.9) | 417 (15.4) | 227 (14.5) | **< 0.001** |
| 2009-2010 | 1,018 (14.7) | 88 (13.9) | 300 (15.5) | 425 (15.5) | 205 (12.7) |  |
| 2011-2012 | 933 (17.7) | 96 (17.8) | 269 (16.5) | 370 (18.5) | 198 (17.3) |  |
| 2013-2014 | 985 (18.1) | 130 (25.7) | 344 (22.5) | 334 (15.8) | 177 (14.4) |  |
| 2015-2016 | 971 (18.5) | 74 (16.9) | 259 (18.8) | 408 (17.1) | 230 (21.1) |  |
| 2017-2018 | 805 (16.1) | 51 (10.9) | 181 (11.9) | 338 (17.6) | 235 (20.0) |  |
| **Frailty** |  |  |  |  |  |  |
| No | 3,639 (68.6) | 229 (50.5) | 856 (57.2) | 1,570 (72.9) | 984 (80.2) | **< 0.001** |
| Yes | 2,087 (31.4) | 295 (49.5) | 782 (42.8) | 722 (27.1) | 288 (19.8) |  |
| Footnotes: Continuous variables are presented as mean ± SE, and categorical variables are presented as n (weighted %). ^a^ *P*-values were assessed by one way ANOVA (continuous variables) or by Chi-square test (categorical variables). *P*-values shown in bold were statistically significant. Abbreviations: BMI, Body mass index; CVDs, Cardiovascular diseases; DM, Diabetes mellitus; MVPA, Moderate-to-vigorous physical activity; PIR, Poverty income ratio; SB, Sedentary behavior; SE, Standard error. | | | | | | |

| **Supplementary Table 4 Baseline characteristics of study participants stratified by age.** | | | | | |
| --- | --- | --- | --- | --- | --- |
|  | **Total participants** | **Age groups** | | | ***P*-value^a^** |
|  |  | **18-39 years** | **40-59 years** | **60-64years** |  |
| **Gender** |  |  |  |  |  |
| Female | 2,956 (52.4) | 547 (49.1) | 1,167 (53.1) | 1,242 (53.5) | 0.2 |
| Male | 2,770 (47.6) | 543 (50.9) | 1,003 (46.9) | 1,224 (46.5) |  |
| **Race/ethnicity** |  |  |  |  |  |
| Non-Hispanic White | 2,435 (71.4) | 553 (67.0) | 1,031 (70.0) | 851 (75.8) | **< 0.001** |
| Non-Hispanic Black | 1,382 (11.3) | 212 (11.3) | 513 (12.8) | 657 (9.7) |  |
| Mexican American | 778 (5.9) | 114 (7.6) | 237 (5.8) | 427 (5.0) |  |
| Other Races | 1,131 (11.3) | 211 (14.1) | 389 (11.5) | 531 (9.6) |  |
| **Education level** |  |  |  |  |  |
| Less than high school | 569 (4.8) | 46 (3.2) | 208 (5.5) | 315 (5.0) | **< 0.001** |
| High school | 2,372 (38.1) | 538 (44.7) | 949 (39.8) | 885 (32.2) |  |
| More than high school | 2,785 (57.1) | 506 (52.1) | 1,013 (54.7) | 1,266 (62.8) |  |
| **PIR** | 2.75 ± 0.05 | 2.16 ± 0.07 | 2.49 ± 0.07 | 3.39 ± 0.06 | **< 0.001** |
| <1.3 | 2,394 (29.7) | 574 (41.4) | 1,096 (35.0) | 724 (16.6) | **< 0.001** |
| 1.3-3.5 | 1,871 (32.2) | 345 (34.7) | 683 (33.2) | 843 (29.5) |  |
| >3.5 | 1,461 (38.1) | 171 (23.9) | 391 (31.8) | 899 (53.8) |  |
| **BMI (kg/m2)** | 30.60 ± 0.16 | 29.93 ± 0.34 | 31.28 ± 0.24 | 30.22 ± 0.23 | **< 0.001** |
| Underweight (<18.5) | 99 (1.9) | 29 (3.1) | 42 (2.2) | 28 (0.8) | **< 0.001** |
| Normal (18.5-25) | 1,207 (22.0) | 304 (30.4) | 407 (18.7) | 496 (20.7) |  |
| Overweight (25-30) | 1,701 (29.5) | 268 (24.6) | 590 (28.9) | 843 (33.1) |  |
| Obese(≥30) | 2,719 (46.6) | 489 (42.0) | 1,131 (50.1) | 1,099 (45.5) |  |
| **Marital status** |  |  |  |  |  |
| Married/Living with Partner | 3,231 (60.9) | 518 (48.2) | 1,145 (58.7) | 1,568 (71.0) | **< 0.001** |
| Never married | 969 (16.9) | 457 (42.7) | 330 (13.2) | 182 (5.5) |  |
| Widowed/Divorced/Separated | 1,526 (22.2) | 115 (9.1) | 695 (28.1) | 716 (23.4) |  |
| **Smoking status** |  |  |  |  |  |
| Never | 2,570 (45.8) | 506 (46.7) | 840 (41.3) | 1,224 (50.4) | **< 0.001** |
| Former | 1,464 (26.5) | 162 (17.6) | 509 (24.6) | 793 (33.9) |  |
| Current | 1,692 (27.7) | 422 (35.7) | 821 (34.1) | 449 (15.6) |  |
| **Alcohol consumption** |  |  |  |  |  |
| Never | 746 (10.2) | 137 (11.3) | 248 (9.2) | 361 (10.8) | **< 0.001** |
| Former | 1,104 (16.3) | 126 (9.8) | 481 (20.0) | 497 (15.9) |  |
| Current | 3,876 (73.5) | 827 (78.9) | 1,441 (70.8) | 1,608 (73.3) |  |
| **Hypertension** |  |  |  |  |  |
| No | 2,612 (50.0) | 794 (74.9) | 953 (46.8) | 865 (38.8) | **< 0.001** |
| Yes | 3,114 (50.0) | 296 (25.1) | 1,217 (53.2) | 1,601 (61.2) |  |
| **DM** |  |  |  |  |  |
| No | 3,780 (71.6) | 925 (86.7) | 1,392 (68.5) | 1,463 (66.1) | **< 0.001** |
| Yes | 1,946 (28.4) | 165 (13.3) | 778 (31.5) | 1,003 (33.9) |  |
| **CVDs** |  |  |  |  |  |
| No | 4,894 (87.7) | 1,018 (94.1) | 1,784 (85.1) | 2,092 (86.8) | **< 0.001** |
| Yes | 832 (12.3) | 72 (5.9) | 386 (14.9) | 374 (13.2) |  |
| **SB time (Minutes/per day)** | 381.59 ± 4.11 | 372.83 ± 8.51 | 388.48 ± 6.51 | 378.97 ± 6.43 | 0.27 |
| **Sleep time** | 7.06 ± 0.03 | 7.26 ± 0.08 | 6.89 ± 0.05 | 7.14 ± 0.03 | **< 0.001** |
| **Total MVPA (Minutes/per week)** | 1,175.71 ± 41.54 | 1,616.01 ± 74.39 | 1,104.20 ± 62.46 | 958.19 ± 53.11 | **< 0.001** |
| **Total energy intake (kcal)** | 2,147.56 ± 16.93 | 2,294.10 ± 36.00 | 2,183.63 ± 30.26 | 2,018.72 ± 21.23 | **< 0.001** |
| **Meeting 24-h movement guidelines** |  |  |  |  |  |
| None | 524 (8.5) | 88 (7.1) | 228 (10.0) | 208 (7.7) | **< 0.001** |
| **Meeting individual guidelines** |  |  |  |  |  |
| MVPA | 386 (7.0) | 94 (8.2) | 153 (7.3) | 139 (6.0) |  |
| SB | 718 (9.2) | 98 (7.3) | 319 (11.9) | 301 (7.2) |  |
| Sleep | 534 (10.2) | 65 (6.4) | 217 (10.8) | 252 (11.9) |  |
| **Meeting specific guideline combinations** |  |  |  |  |  |
| SB + Sleep | 762 (12.1) | 87 (7.6) | 281 (11.8) | 394 (15.1) |  |
| MVPA + Sleep | 1,035 (17.3) | 253 (22.8) | 388 (17.5) | 394 (13.7) |  |
| MVPA + SB | 495 (11.8) | 116 (12.5) | 166 (11.0) | 213 (12.3) |  |
| **Number of guidelines met** |  |  |  |  |  |
| 0 | 524 (8.5) | 88 (7.1) | 228 (10.0) | 208 (7.7) | **< 0.001** |
| 1 | 1,638 (26.4) | 257 (21.9) | 689 (30.0) | 692 (25.1) |  |
| 2 | 2,292 (41.2) | 456 (42.9) | 835 (40.4) | 1,001 (41.0) |  |
| 3 | 1,272 (23.8) | 289 (28.1) | 418 (19.6) | 565 (26.2) |  |
| **Frailty** |  |  |  |  |  |
| No | 3,639 (68.6) | 795 (77.0) | 1,024 (54.2) | 1,820 (80.0) | **< 0.001** |
| Yes | 2,087 (31.4) | 295 (23.0) | 1,146 (45.8) | 646 (20.0) |  |
| **Year cycle** |  |  |  |  |  |
| 2007-2008 | 1,014 (15.0) | 195 (15.9) | 355 (15.2) | 464 (14.2) | 0.5 |
| 2009-2010 | 1,018 (14.7) | 181 (13.5) | 408 (15.0) | 429 (15.1) |  |
| 2011-2012 | 933 (17.7) | 168 (15.2) | 349 (17.3) | 416 (19.5) |  |
| 2013-2014 | 985 (18.1) | 188 (16.7) | 389 (18.5) | 408 (18.3) |  |
| 2015-2016 | 971 (18.5) | 196 (19.6) | 379 (18.9) | 396 (17.3) |  |
| 2017-2018 | 805 (16.1) | 162 (19.1) | 290 (14.9) | 353 (15.5) |  |
| Footnotes: Continuous variables are presented as weighted mean ± SE, and categorical variables are presented as n (weighted %). ^a^ *P*-values were assessed by one way ANOVA (continuous variables) or by Chi-square test (categorical variables). P-values shown in bold were statistically significant. Abbreviations: BMI, Body mass index; CVDs, Cardiovascular diseases; DM, Diabetes mellitus; MVPA, Moderate-to-vigorous physical activity; PIR, Poverty income ratio; SB, Sedentary behavior; SE, Standard error. | | | | | |

| **Supplementary Table 5 Baseline characteristics of study participants stratified by gender.** | | | | |
| --- | --- | --- | --- | --- |
|  | **Total participants** | **Gender** | | ***P*-value^a^** |
|  |  | **Female** | **Male** |  |
| **Age** | 50.25 ± 0.31 | 50.57 ± 0.37 | 49.90 ± 0.34 | 0.07 |
| 18-39 | 1,090 (21.9) | 547 (20.5) | 543 (23.4) | 0.2 |
| 40-59 | 2,170 (41.6) | 1,167 (42.2) | 1,003 (41.0) |  |
| 60-64 | 2,466 (36.5) | 1,242 (37.3) | 1,224 (35.7) |  |
| **Race/ethnicity** |  |  |  |  |
| Non-Hispanic White | 2,435 (71.4) | 1,251 (70.8) | 1,184 (72.1) | 0.1 |
| Non-Hispanic Black | 1,382 (11.3) | 711 (12.2) | 671 (10.3) |  |
| Mexican American | 778 (5.9) | 394 (5.5) | 384 (6.3) |  |
| Other Races | 1,131 (11.3) | 600 (11.5) | 531 (11.2) |  |
| **Education level** |  |  |  |  |
| Less than high school | 569 (4.8) | 267 (4.4) | 302 (5.3) | 0.03 |
| High school | 2,372 (38.1) | 1,172 (36.6) | 1,200 (39.8) |  |
| More than high school | 2,785 (57.1) | 1,517 (59.0) | 1,268 (55.0) |  |
| **PIR** | 2.75 ± 0.05 | 2.67 ± 0.06 | 2.83 ± 0.06 | 0.01 |
| <1.3 | 2,394 (29.7) | 1,288 (31.4) | 1,106 (27.9) | **< 0.05** |
| 1.3-3.5 | 1,871 (32.2) | 945 (32.0) | 926 (32.3) |  |
| >3.5 | 1,461 (38.1) | 723 (36.6) | 738 (39.8) |  |
| **BMI (kg/m2)** | 30.60 ± 0.16 | 31.42 ± 0.22 | 29.69 ± 0.18 | **< 0.001** |
| Underweight (<18.5) | 99 (1.9) | 58 (2.5) | 41 (1.2) | **< 0.001** |
| Normal (18.5-25) | 1,207 (22.0) | 552 (21.0) | 655 (23.1) |  |
| Overweight (25-30) | 1,701 (29.5) | 760 (25.9) | 941 (33.4) |  |
| Obese (≥30) | 2,719 (46.6) | 1,586 (50.6) | 1,133 (42.3) |  |
| **Marital status** |  |  |  |  |
| Married/Living with Partner | 3,231 (60.9) | 1,521 (58.4) | 1,710 (63.6) | **< 0.001** |
| Never married | 969 (16.9) | 482 (14.7) | 487 (19.3) |  |
| Widowed/Divorced/Separated | 1,526 (22.2) | 953 (26.9) | 573 (17.1) |  |
| **Smoking status** |  |  |  |  |
| Never | 2,570 (45.8) | 1,523 (49.6) | 1,047 (41.6) | **< 0.001** |
| Former | 1,464 (26.5) | 636 (23.7) | 828 (29.6) |  |
| Current | 1,692 (27.7) | 797 (26.7) | 895 (28.8) |  |
| **Alcohol consumption** |  |  |  |  |
| Never | 746 (10.2) | 533 (12.6) | 213 (7.7) | **< 0.001** |
| Former | 1,104 (16.3) | 554 (16.4) | 550 (16.2) |  |
| Current | 3,876 (73.5) | 1,869 (71.0) | 2,007 (76.2) |  |
| **Hypertension** |  |  |  |  |
| No | 2,612 (50.0) | 1,352 (51.1) | 1,260 (48.9) | 0.2 |
| Yes | 3,114 (50.0) | 1,604 (48.9) | 1,510 (51.1) |  |
| **DM** |  |  |  |  |
| No | 3,780 (71.6) | 1,984 (73.6) | 1,796 (69.5) | **< 0.05** |
| Yes | 1,946 (28.4) | 972 (26.4) | 974 (30.5) |  |
| **CVDs** |  |  |  |  |
| No | 4,894 (87.7) | 2,579 (89.2) | 2,315 (86.0) | **< 0.05** |
| Yes | 832 (12.3) | 377 (10.8) | 455(14.0) |  |
| **SB time (Minutes/per day)** | 381.59 ± 4.11 | 379.59 ± 5.22 | 383.78±5.82 | 0.57 |
| **Sleep time** | 7.06 ± 0.03 | 7.10 ± 0.04 | 7.03±0.04 | 0.22 |
| **Total MVPA (Minutes/per week)** | 1,175.71 ± 41.54 | 968.17 ± 57.48 | 1,384.03±56.05 | **< 0.001** |
| **Total energy intake (kcal)** | 2,147.56 ± 16.93 | 1,820.77 ± 17.11 | 2,506.96 ± 26.36 | **< 0.001** |
| **Meeting 24-h movement guidelines** |  |  |  |  |
| None | 524 (8.5) | 279 (9.1) | 245 (8.0) | **< 0.001** |
| **Meeting individual guidelines** |  |  |  |  |
| MVPA | 386 (7.0) | 181 (6.2) | 205 (7.9) |  |
| SB | 718 (9.2) | 427 (10.3) | 291 (7.9) |  |
| Sleep | 534 (10.2) | 326 (11.9) | 208 (8.4) |  |
| **Meeting specific guideline combinations** |  |  |  |  |
| SB + Sleep | 762 (12.1) | 459 (14.6) | 303 (9.3) |  |
| MVPA + Sleep | 1,035 (17.3) | 459 (15.5) | 576 (19.2) |  |
| MVPA + SB | 495 (11.8) | 234 (10.2) | 261 (13.5) |  |
| **Number of guidelines met** |  |  |  |  |
| 0 | 524 (8.5) | 279 (9.1) | 245 (8.0) | 0.03 |
| 1 | 1,638 (26.4) | 934 (28.5) | 704 (24.2) |  |
| 2 | 2,292 (41.2) | 1,152 (40.3) | 1,140 (42.1) |  |
| 3 | 1,272 (23.8) | 591 (22.1) | 681 (25.8) |  |
| **Frailty** |  |  |  |  |
| No | 3,639 (68.6) | 1,711 (62.8) | 1,928 (74.9) | **< 0.001** |
| Yes | 2,087 (31.4) | 1,245 (37.2) | 842 (25.1) |  |
| **Year cycle** |  |  |  |  |
| 2007-2008 | 1,014 (15.0) | 520 (15.0) | 494 (15.0) | 0.4 |
| 2009-2010 | 1,018 (14.7) | 535 (15.1) | 483 (14.3) |  |
| 2011-2012 | 933 (17.7) | 475 (17.1) | 458 (18.3) |  |
| 2013-2014 | 985 (18.1) | 542 (19.1) | 443 (16.9) |  |
| 2015-2016 | 971 (18.5) | 484 (17.9) | 487 (19.1) |  |
| 2017-2018 | 805 (16.1) | 400 (15.8) | 405 (16.4) |  |
| Footnotes: Continuous variables are presented as weighted mean ± SE, and categorical variables are presented as n (weighted %). ^a^ *P*-values were assessed by T-test (continuous variables) or by Chi-square test (categorical variables). P-values shown in bold were statistically significant. Abbreviations: BMI, Body mass index; CVDs, Cardiovascular diseases; DM, Diabetes mellitus; MVPA, Moderate-to-vigorous physical activity; PIR, Poverty income ratio; SB, Sedentary behavior; SE, Standard error. | | | | |

| **Supplementary Table 6 Baseline characteristics of study participants stratified by race/ethnicity.** | | | | | | |
| --- | --- | --- | --- | --- | --- | --- |
|  | **Total participants** | **Race/ethnicity** | | | | ***P*-value^a^** |
|  |  | **Non-Hispanic White** | **Non-Hispanic Black** | **Mexican American** | **Other Race** |  |
| **Age** | 50.25 ± 0.31 | 50.90 ± 0.39 | 49.59 ± 0.47 | 47.76 ± 0.56 | 48.10 ± 0.54 | **< 0.001** |
| 18-39 | 1090 (21.9) | 553 (20.5) | 212 (21.8) | 114 (28.3) | 211 (27.2) | **< 0.001** |
| 40-59 | 2170 (41.6) | 1031 (40.8) | 513 (47.1) | 237 (40.6) | 389 (42.0) |  |
| 60-64 | 2466 (36.5) | 851 (38.7) | 657 (31.2) | 427 (31.1) | 531 (30.8) |  |
| **Gender** |  |  |  |  |  |  |
| Female | 2956 (52.4) | 1251 (51.9) | 711 (56.5) | 394 (48.9) | 600 (52.9) | 0.1 |
| Male | 2770 (47.6) | 1184 (48.1) | 671 (43.5) | 384 (51.1) | 531 (47.1) |  |
| **Education level** |  |  |  |  |  |  |
| Less than high school | 569 (4.8) | 101 (2.5) | 51 (3.1) | 268 (27.1) | 149 (9.3) | **< 0.001** |
| High school | 2372 (38.1) | 1004 (36.2) | 676 (49.7) | 302 (42.6) | 390 (36.4) |  |
| More than high school | 2785 (57.1) | 1330 (61.3) | 655 (47.2) | 208 (30.4) | 592 (54.3) |  |
| **PIR** | 2.75 ± 0.05 | 3.03 ± 0.07 | 1.95 ± 0.07 | 1.83 ± 0.07 | 2.25 ± 0.08 | **< 0.001** |
| <1.3 | 2394 (29.7) | 995 (24.4) | 585 (45.4) | 358 (46.7) | 456 (38.9) | **< 0.001** |
| 1.3-3.5 | 1871 (32.2) | 699 (30.5) | 489 (35.4) | 300 (38.9) | 383 (35.9) |  |
| >3.5 | 1461 (38.1) | 741 (45.1) | 308 (19.2) | 120 (14.4) | 292 (25.2) |  |
| **BMI (kg/m2)** | 30.60 ± 0.16 | 30.31 ± 0.21 | 32.50 ± 0.27 | 31.56 ± 0.36 | 30.04 ± 0.33 | **< 0.001** |
| Underweight (<18.5) | 99 (1.9) | 47 (1.9) | 29 (1.9) | 2 (0.5) | 21 (2.4) | **< 0.05** |
| Normal (18.5-25) | 1207 (22.0) | 540 (22.7) | 268 (18.3) | 109(15.2) | 290 (24.6) |  |
| Overweight (25-30) | 1701 (29.5) | 711 (30.1) | 362 (25.5) | 264 (32.8) | 364 (27.9) |  |
| Obese (≥30) | 2719 (46.6) | 1137 (45.3) | 723 (54.3) | 403 (51.5) | 456 (45.1) |  |
| **Marital status** |  |  |  |  |  |  |
| Married/Living with Partner | 3231 (60.9) | 1415 (63.7) | 590 (40.2) | 520 (64.9) | 706 (61.6) | **< 0.001** |
| Never married | 969 (16.9) | 378 (14.7) | 346 (29.7) | 80 (14.7) | 165 (18.5) |  |
| Widowed/Divorced/Separated | 1526 (22.2) | 642 (21.5) | 446 (30.1) | 178 (20.4) | 260 (19.9) |  |
| **Smoking status** |  |  |  |  |  |  |
| Never | 2570 (45.8) | 908 (44.3) | 630 (47.2) | 432 (53.4) | 600 (50.0) | **< 0.001** |
| Former | 1464 (26.5) | 675 (28.4) | 295 (18.6) | 206 (24.2) | 288 (23.3) |  |
| Current | 1692 (27.7) | 852 (27.3) | 457 (34.2) | 140 (22.4) | 243 (26.7) |  |
| **Alcohol consumption** |  |  |  |  |  |  |
| Never | 746 (10.2) | 201 (8.1) | 179 (13.4) | 142 (15.2) | 224 (17.6) | **< 0.001** |
| Former | 1104 (16.3) | 502 (16.2) | 268 (16.8) | 155 (17.4) | 179 (15.4) |  |
| Current | 3876 (73.5) | 1732 (75.6) | 935 (69.8) | 481 (67.4) | 728 (67.0) |  |
| **Hypertension** |  |  |  |  |  |  |
| No | 2612 (50.0) | 1222 (51.7) | 439 (35.3) | 374 (56.4) | 577 (51.1) | **< 0.001** |
| Yes | 3114 (50.0) | 1213 (48.3) | 943 (64.7) | 404 (43.6) | 554 (48.9) |  |
| **DM** |  |  |  |  |  |  |
| No | 3780 (71.6) | 1751 (73.8) | 861 (65.0) | 456 (65.3) | 712 (67.8) | **< 0.001** |
| Yes | 1946 (28.4) | 684 (26.2) | 521 (35.0) | 322 (34.7) | 419 (32.2) |  |
| **CVDs** |  |  |  |  |  |  |
| No | 4894 (87.7) | 2090 (88.7) | 1121 (81.1) | 685 (89.2) | 998 (87.2) | **< 0.001** |
| Yes | 832 (12.3) | 345 (11.3) | 261 (18.9) | 93 (10.8) | 133 (12.8) |  |
| **SB time (Minutes/per day)** | 381.59 ± 4.11 | 395.35 ± 5.51 | 375.39 ± 8.11 | 305.30 ± 12.04 | 340.72 ± 8.40 | **< 0.001** |
| **Sleep time** | 7.06 ± 0.03 | 7.11 ± 0.05 | 6.69 ± 0.07 | 7.18 ± 0.10 | 7.08 ± 0.07 | **< 0.001** |
| **Total MVPA (Minutes/per week)** | 1175.71 ± 41.54 | 1158.02 ± 54.05 | 1077.19 ± 54.79 | 1461.69 ± 120.84 | 1250.32 ± 91.41 | 0.02 |
| **Total energy intake (kcal)** | 2147.56 ± 16.93 | 2178.47 ± 20.36 | 2071.06 ± 36.16 | 2178.21 ± 50.91 | 2013.28 ± 46.25 | **< 0.05** |
| **Meeting 24-h movement guidelines** |  |  |  |  |  |  |
| None | 524 (8.5) | 242 (8.4) | 149 (11.2) | 40 (6.5) | 93 (8.0) | **< 0.001** |
| **Meeting individual guidelines** |  |  |  |  |  |  |
| MVPA | 386 (7.0) | 172 (7.1) | 126 (10.3) | 30 (4.2) | 58 (4.8) |  |
| SB | 718 (9.2) | 242 (7.6) | 208 (14.3) | 124 (14.8) | 144 (11.4) |  |
| Sleep | 534 (10.2) | 257 (10.8) | 131 (9.1) | 58 (7.6) | 88 (9.0) |  |
| **Meeting specific guideline combinations** |  |  |  |  |  |  |
| SB + Sleep | 762 (12.1) | 271 (11.4) | 167 (11.7) | 154 (17.1) | 170 (14.4) |  |
| MVPA + Sleep | 1035 (17.3) | 416 (16.6) | 282 (20.6) | 131 (16.7) | 206 (18.4) |  |
| MVPA + SB | 495 (11.8) | 281 (13.8) | 87 (6.3) | 37 (6.0) | 90 (7.6) |  |
| **Number of guidelines met** |  |  |  |  |  |  |
| 0 | 524 (8.5) | 242 (8.4) | 149 (11.2) | 40 (6.5) | 93 (8.0) | **< 0.001** |
| 1 | 1638 (26.4) | 671 (25.5) | 465 (33.7) | 212 (26.6) | 290 (25.2) |  |
| 2 | 2292 (41.2) | 968 (41.8) | 536 (38.5) | 322 (39.8) | 466 (40.4) |  |
| 3 | 1272 (23.8) | 554 (24.3) | 232 (16.6) | 204 (27.0) | 282 (26.4) |  |
| **Frailty** |  |  |  |  |  |  |
| No | 3639 (68.6) | 1560 (71.4) | 797 (54.9) | 526 (69.8) | 756 (64.0) | **< 0.001** |
| Yes | 2087 (31.4) | 875 (28.6) | 585 (45.1) | 252 (30.2) | 375 (36.0) |  |
| **Year cycle** |  |  |  |  |  |  |
| 2007-2008 | 1014 (15.0) | 488 (15.3) | 235 (14.8) | 146 (14.5) | 145 (13.5) | 0.9 |
| 2009-2010 | 1018 (14.7) | 485 (14.8) | 217 (17.4) | 177 (16.8) | 139 (10.6) |  |
| 2011-2012 | 933 (17.7) | 374 (18.2) | 287 (17.6) | 81 (13.9) | 191 (16.6) |  |
| 2013-2014 | 985 (18.1) | 461 (18.3) | 230 (18.3) | 112 (17.7) | 182 (16.6) |  |
| 2015-2016 | 971 (18.5) | 338 (18.1) | 214 (17.0) | 167 (20.5) | 252 (21.6) |  |
| 2017-2018 | 805 (16.1) | 289 (15.4) | 199 (14.9) | 95 (16.6) | 222 (21.1) |  |
| Footnotes: Continuous variables are presented as weighted mean ± SE, and categorical variables are presented as n (weighted %). ^a^ *P*-values were assessed by one way ANOVA (continuous variables) or by Chi-square test (categorical variables). *P*-values shown in bold were statistically significant. Abbreviations: BMI, Body mass index; CVDs, Cardiovascular diseases; DM, Diabetes mellitus; MVPA, Moderate-to-vigorous physical activity; PIR, Poverty income ratio; SB, Sedentary behavior; SE, Standard error. | | | | | | |

| **Supplementary Table 7 Associations of meeting 24-h movement guidelines with frailty index.** | | | | | | | | |
| --- | --- | --- | --- | --- | --- | --- | --- | --- |
| **Meeting 24-h movement guidelines** | **Crude Model** | |  | **Model 1** | |  | **Model 2** | |
|  | ***β*-coefficient (95% CI)** | ***P*-value** |  | ***β*-coefficient (95% CI)** | ***P*-value** |  | ***β*-coefficient (95% CI)** | ***P*-value** |
| None | Reference | |  | Reference | |  | Reference | |
| **Meeting individual guidelines** |  |  |  |  |  |  |  |  |
| MVPA | **-0.056 (-0.077, -0.036)** | **< 0.001** |  | **-0.053 (-0.074, -0.033)** | **< 0.001** |  | **-0.031(-0.049, -0.014)** | **< 0.001** |
| SB | -0.006 (-0.026, 0.015) | 0.580 |  | -0.008 (-0.028, 0.012) | 0.435 |  | -0.004(-0.023, 0.015) | 0.672 |
| Sleep | **-0.046 (-0.064, -0.028)** | **< 0.001** |  | **-0.046 (-0.064, -0.028)** | **< 0.001** |  | **-0.029(-0.045, -0.013)** | **< 0.001** |
| **Meeting specific guideline combinations** |  |  |  |  |  |  |  |  |
| Sleep + SB | **-0.056 (-0.075, -0.036)** | **< 0.001** |  | **-0.057 (-0.076, -0.038)** | **< 0.001** |  | **-0.04 (-0.058, -0.021)** | **< 0.001** |
| MVPA + Sleep | **-0.099 (-0.117, -0.080)** | **< 0.001** |  | **-0.092 (-0.110, -0.075)** | **< 0.001** |  | **-0.054 (-0.071, -0.036)** | **< 0.001** |
| MVPA + SB | **-0.069 (-0.090, -0.049)** | **< 0.001** |  | **-0.066 (-0.086, -0.046)** | **< 0.001** |  | **-0.049 (-0.068, -0.031)** | **< 0.001** |
| **Number of guidelines met** |  |  |  |  |  |  |  |  |
| 1 | **-0.035 (-0.052, -0.017)** | **< 0.001** |  | **-0.035 (-0.052, -0.018)** | **< 0.001** |  | **-0.021 (-0.037, -0.005)** | **< 0.05** |
| 2 | **-0.074 (-0.092, -0.056)** | **< 0.001** |  | **-0.071 (-0.089, -0.053)** | **< 0.001** |  | **-0.047 (-0.064, -0.031)** | **< 0.001** |
| 3 | **-0.097 (-0.114, -0.080)** | **< 0.001** |  | **-0.093 (-0.110, -0.077)** | **< 0.001** |  | **-0.065 (-0.081, -0.048)** | **< 0.001** |
| *P* for trend |  | **< 0.001** |  |  | **< 0.001** |  |  | **< 0.001** |
| Footnotes: The results of β-coefficient (95% CI), and *P*-value shown in bold were statistically significant. The multivariable model was adjusted for gender, race/ethnicity, education level, marital status, PIR, BMI, smoking, alcohol consumption and total energy intake. Abbreviations: BMI = body mass index; CI = confidence interval; MVPA = moderate-to-vigorous physical activity; PIR = poverty income ratio; SB = sedentary behavior. | | | | | | | | |

| **Supplementary Table 8 Further adjustments in sensitivity analyses for associations of meeting 24-h movement guidelines with frailty.** | | | | | | | | | | | | | | |
| --- | --- | --- | --- | --- | --- | --- | --- | --- | --- | --- | --- | --- | --- | --- |
| **Meeting 24-h movement guidelines** | **Adjusted for survey cycles** | |  | **Adjusted for HEI-2015** | |  | **Adjusted for CVDs** | |  | **Adjusted for Hypertension** | |  | **Adjusted for DM** | |
|  | **AOR (95% CI)** | ***P*-value** |  | **AOR (95% CI)** | ***P*-value** |  | **AOR (95% CI)** | ***P*-value** |  | **AOR (95% CI)** | ***P*-value** |  | **AOR (95% CI)** | ***P*-value** |
| None | Reference | |  | Reference | |  | Reference | |  | Reference | |  | Reference | |
| **Meeting individual guidelines** |  |  |  |  |  |  |  |  |  |  |  |  |  |  |
| MVPA | 0.772 (0.536, 1.113) | 0.162 |  | 0.786 (0.545, 1.132) | 0.293 |  | 0.793 (0.549, 1.144) | 0.210 |  | 0.851 (0.595, 1.218) | 0.372 |  | 0.791 (0.551, 1.133) | 0.197 |
| SB | 1.186 (0.804, 1.750) | 0.384 |  | 1.159 (0.785, 1.710) | 0.559 |  | 1.230 (0.851, 1.777) | 0.267 |  | 1.282 (0.867, 1.895) | 0.209 |  | 1.174 (0.775, 1.778) | 0.443 |
| Sleep | 0.918 (0.622, 1.353) | 0.66 |  | 0.915 (0.615, 1.362) | 0.970 |  | 0.944 (0.649, 1.373) | 0.759 |  | 0.984 (0.665, 1.456) | 0.935 |  | 0.895 (0.593, 1.350) | 0.591 |
| **Meeting specific guideline combinations** |  |  |  |  |  |  |  |  |  |  |  |  |  |  |
| Sleep + SB | **0.611 (0.420, 0.890)** | **< 0.05** |  | **0.612 (0.423, 0.885)** | **< 0.05** |  | **0.644 (0.447, 0.928)** | **< 0.05** |  | **0.635 (0.440, 0.917)** | **< 0.05** |  | **0.624 (0.418, 0.930)** | **< 0.05** |
| MVPA + Sleep | **0.379 (0.249, 0.575)** | **< 0.001** |  | **0.390 (0.257, 0.594)** | **< 0.001** |  | **0.395 (0.261, 0.598)** | **< 0.001** |  | **0.435 (0.285, 0.664)** | **< 0.001** |  | **0.402 (0.262, 0.616)** | **< 0.001** |
| MVPA + SB | **0.558 (0.384, 0.809)** | **< 0.05** |  | **0.557 (0.384, 0.808)** | **< 0.05** |  | **0.584 (0.400, 0.852)** | **< 0.05** |  | **0.598 (0.414, 0.865)** | **< 0.05** |  | **0.580 (0.395, 0.851)** | **< 0.05** |
| **Number of guidelines met** |  |  |  |  |  |  |  |  |  |  |  |  |  |  |
| 1 | 0.963 (0.698, 1.328) | 0.815 |  | 0.959 (0.692, 1.329) | 0.799 |  | 0.993 (0.731, 1.349) | 0.966 |  | 1.044 (0.757, 1.440) | 0.791 |  | 0.955 (0.682, 1.339) | 0.788 |
| 2 | **0.523 (0.374, 0.730)** | **< 0.001** |  | **0.528 (0.378, 0.737)** | **< 0.001** |  | **0.549 (0.396, 0.762)** | **< 0.05** |  | **0.566 (0.408, 0.786)** | **< 0.001** |  | **0.544 (0.385, 0.768)** | **< 0.001** |
| 3 | **0.370 (0.258, 0.529)** | **< 0.001** |  | **0.378 (0.265, 0.540)** | **< 0.001** |  | **0.384 (0.274, 0.538)** | **< 0.001** |  | **0.425 (0.296, 0.608)** | **< 0.001** |  | **0.406 (0.281, 0.589)** | **< 0.001** |
| *P* for trend |  | **< 0.001** |  |  | **< 0.001** |  |  | **< 0.001** |  |  | **< 0.001** |  |  | **< 0.001** |
| Footnotes: The results of AOR (95% CI), and *P*-value shown in bold were statistically significant. Sensitivity analyses were performed based on Model 2, and plus adjusted by adding variables including survey cycles, DM, CVD, CKD, Hypertension and HEI-2015, respectively. Abbreviations: AOR, Adjusted odds ratio; BMI, Body mass index; CI, Confidence interval; CVDs, Cardiovascular diseases; DM, Diabetes mellitus; HEI-2015, Healthy eating index-2015; MVPA, Moderate-to-vigorous physical activity; PIR, Poverty income ratio; SB, Sedentary behavior. | | | | | | | | | | | | | | |
