# Supplementary figures and images for: Is meeting 24-hour movement guidelines associated with a lower risk of frailty among adults?
Source: Int J Behav Nutr Phys Act. 2025 Feb 21;22:21. doi: 10.1186/s12966-025-01722-x (PMC11846395; doi:10.1186/s12966-025-01722-x)

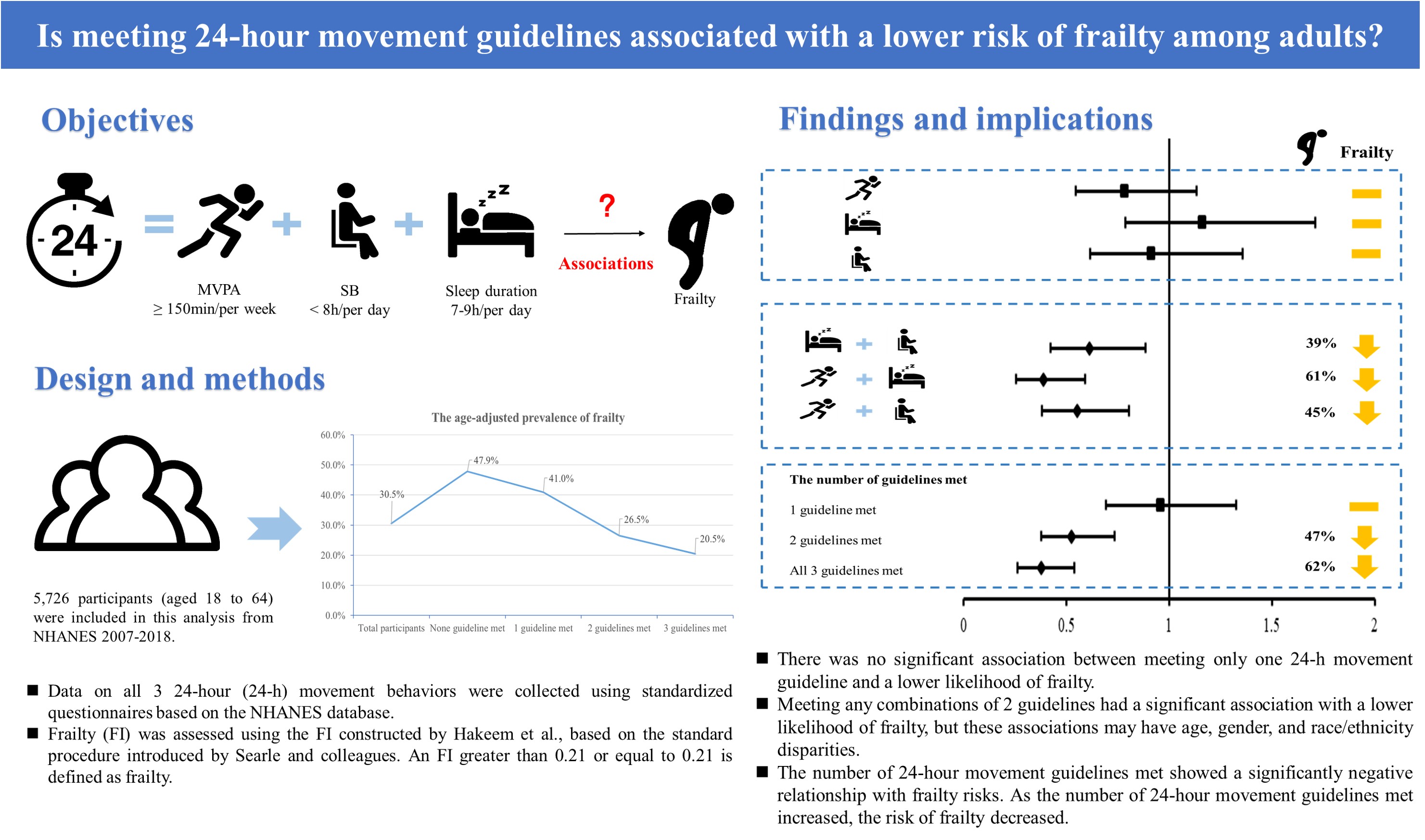

Supplement: Supplementary file 3 — Supplementary Material 3: Graphical abstract [file 12966_2025_1722_MOESM3_ESM.jpg]
